# Ligand-Based Competition Binding by Real-Time ^19^F NMR in Human Cells

**DOI:** 10.1021/acs.jmedchem.3c01600

**Published:** 2024-01-12

**Authors:** Enrico Luchinat, Letizia Barbieri, Ben Davis, Paul A. Brough, Matteo Pennestri, Lucia Banci

**Affiliations:** † Dipartimento di Scienze e Tecnologie Agro-Alimentari, Alma Mater Studiorum—Università di Bologna, Piazza Goidanich 60, Cesena 47521, Italy; ‡ Consorzio Interuniversitario Risonanze Magnetiche di Metallo Proteine—CIRMMP, Via Luigi Sacconi 6, Sesto Fiorentino 50019, Italy; § Vernalis Research, Granta Park, Great Abington, Cambridge CB21 6GB, U.K.; ∥ Pharmaceutical Business Unit, Bruker UK Limited, Banner Lane, Coventry CV4 9GH, U.K.; ⊥ Centro di Risonanze Magnetiche—CERM, Università degli Studi di Firenze, Via Luigi Sacconi 6, Sesto Fiorentino 50019, Italy; # Dipartimento di Chimica, Università degli Studi di Firenze, Via della Lastruccia 3, Sesto Fiorentino 50019, Italy

## Abstract

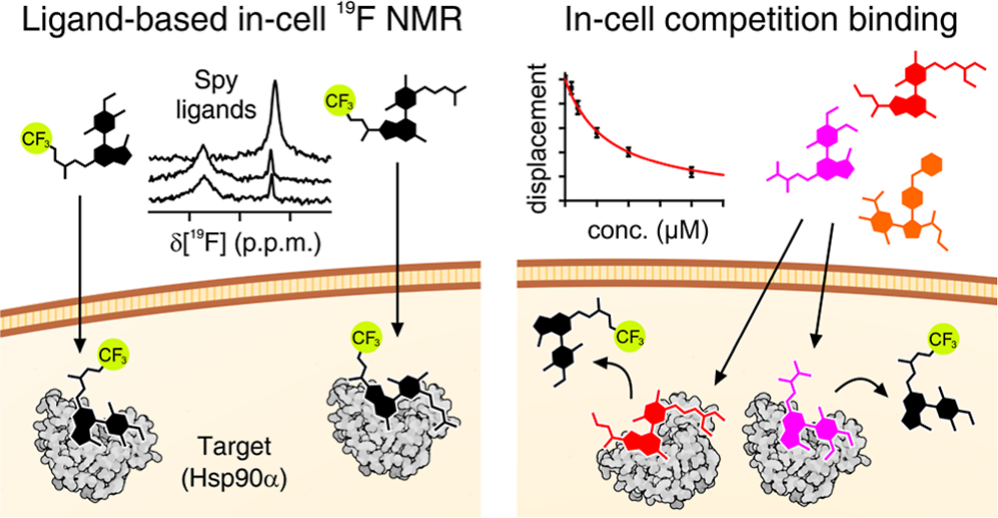

The development of more effective drugs requires knowledge of their bioavailability and binding efficacy directly in the native cellular environment. In-cell nuclear magnetic resonance (NMR) spectroscopy is a powerful tool for investigating ligand–target interactions directly in living cells. However, the target molecule may be NMR-invisible due to interactions with cellular components, while observing the ligand by ^1^H NMR is impractical due to the cellular background. Such limitations can be overcome by observing fluorinated ligands by ^19^F in-cell NMR as they bind to the intracellular target. Here we report a novel approach based on real-time in-cell ^19^F NMR that allows measuring ligand binding affinities in human cells by competition binding, using a fluorinated compound as a reference. The binding of a set of compounds toward Hsp90α was investigated. In principle, this approach could be applied to other pharmacologically relevant targets, thus aiding the design of more effective compounds in the early stages of drug development.

## Introduction

Structure-based drug design relies on the structural knowledge of the target protein to develop novel drugs. Typically, the first steps are performed in vitro, in which molecules binding the target are identified from compound libraries using various screening techniques. The hits from this process are chemically modified, aided by structural information, to find lead candidates with good binding affinity toward the target. Lead compounds are then optimized to improve their activity and drug-like properties both in solution and in cell-based assays. The compounds with the most appropriate profile are promoted to the preclinical phases of development, which include testing efficacy in in vivo disease models before proceeding to clinical trials. At this stage, many promising compounds are discarded despite being active in vitro due to their lack of activity in vivo. Additionally, drug candidates may still fail during the final and most important steps, the clinical trials, making the whole drug development process risky and time- and cost-intensive.

Reducing the attrition rate of drug candidates in later phases of testing remains an important challenge. This is potentially achieved by gaining more knowledge on how the ligand interacts with the target in its physiological environment. In-cell nuclear magnetic resonance (NMR) stands out as a very promising approach to combining the high molecular sensitivity of NMR, which is typically applied to isolated molecules, with the high biological relevance of the cellular context.^1^ Indeed, in-cell NMR can gain structural and functional insights on intracellular macromolecules, including folding and cofactor binding, chemical modification, and interactions with other macromolecules or with external ligands.^2−9^ In particular, in-cell NMR is able to monitor the interaction of ligands or peptides with intracellular macromolecules, i.e., proteins and DNA/RNA.^10−14^ In addition to being able to screen compounds for cell permeability and/or binding to the intracellular target, NMR can also characterize intracellular ligand–target interactions in more detail. NMR bioreactors enable continuous measurement on a sample of viable cells^15−18^ and can be applied to monitor ligand binding in real time, providing information on the kinetics of membrane diffusion and on the binding selectivity toward the target.^19−21^ Furthermore, this approach can be adapted to characterize binding thermodynamics: by employing a reference ligand with a known dissociation constant (*K*_d_), competition binding experiments can be performed in the NMR bioreactor, allowing quantitative measurement of the *K*_d_s of test ligands in the nanomolar range.^22^

All of the above rely on the observation of signals from the target, which is, with few exceptions, made possible by selectively labeling the target molecule with NMR-active isotopes. Despite the huge potential, the broad application of such target-observe approaches is hampered by the fact that the target may interact with other components in the cell. Such interactions are detrimental when observing proteins in solution NMR as they contribute to slowing down molecular tumbling, causing an increase in the transverse spin relaxation rates, which leads to the loss of signal in the NMR spectra.^23−26^ A possible solution to this issue is to observe the ligand instead of the target macromolecule. Ligands are smaller in size, and even after forming a complex with their target, they often retain fast internal motions, which lead to favorable NMR relaxation properties. Typical ligand-observed NMR experiments are based on ^1^H detection, which makes the approach impractical due to the strong interference from other cellular ^1^H signals. Ligand-observed ^1^H NMR often relies on magnetization transfer techniques, such as saturation transfer difference or the transferred nuclear Overhauser effect, and is typically applied to study ligand-membrane receptor interactions in so-called “on-cell NMR” approaches.^27−32^ Such approaches rely upon the fast exchange between free and protein-bound molecules and can be applied either in direct mode to low-affinity ligands or, with ligands in the high nM range, in competition mode using a suitable reference ligand in fast exchange.

Isotopic labeling of the ligand, while possible in principle, would require ad-hoc synthesis of each screened compound starting from enriched precursors. Fluorine represents an interesting exception, as it is commonly found in library compounds and is often introduced to increase affinity and improve physicochemical properties during lead optimization.^33^ The spin-1/2 ^19^F nucleus is optimal for NMR spectroscopy, thanks to its 100% isotopic abundance and high gyromagnetic ratio. In vitro, ^19^F is increasingly employed in fragment and ligand screening by both ligand- and protein-observed NMR.^34,35^ Furthermore, fluorine is natively absent from biological systems and therefore provides virtually background-free ^19^F NMR spectra. ^19^F NMR has been previously applied to observe fluorinated proteins and nucleic acids in living cells.^36−39^ The ^19^F nucleus was also employed as a probe to measure the enzymatic activity of an intracellular target^40^ and to monitor ligand binding to a native protein target in red blood cells, showing great potential in the investigation of protein–ligand interactions in cells.^41^

Here, we provide the first in-cell NMR investigation of fluorinated ligands as they bind to a protein target expressed in cultured human cells. We focused on the N-terminal ATP-binding domain of the human stress-inducible 90 kDa heat shock protein alpha (Hsp90α).^42−44^ Hsp90α is a cytosolic isoform of Hsp90, a homodimeric molecular chaperone that binds and folds other proteins into their functional 3-dimensional structures. Hsp90α expression is induced in cells undergoing proteotoxic stress, where it interacts with a vast number of tumor-promoting proteins. Its role in the cellular adaptation to stress makes Hsp90α a promising drug target. Hsp90α inhibitors inactivate the protein by replacing ATP in the N-terminal domain, resulting in the regulated ubiquitination and proteasome-mediated degradation of its client proteins. In this work, the N-terminal domain of Hsp90α (henceforth Hsp90_N_) was overexpressed in HEK293T cells, where it interacts with the environment, preventing classical protein-observed NMR analysis. The cells were then treated with fluorinated ligands, and their binding to Hsp90_N_ was observed by ^19^F NMR. Finally, competition binding between a fluorinated reference “spy” ligand and other, nonfluorinated ligands was monitored in real-time by ^19^F NMR in the bioreactor to quantitatively measure their intracellular *K*_d_s. This approach is broadly applicable to NMR-invisible targets and will allow optimizing the potency of lead compounds in a more physiological environment, thereby increasing their success rate in later preclinical and clinical tests.

## Results and Discussion

**Chart 1:**
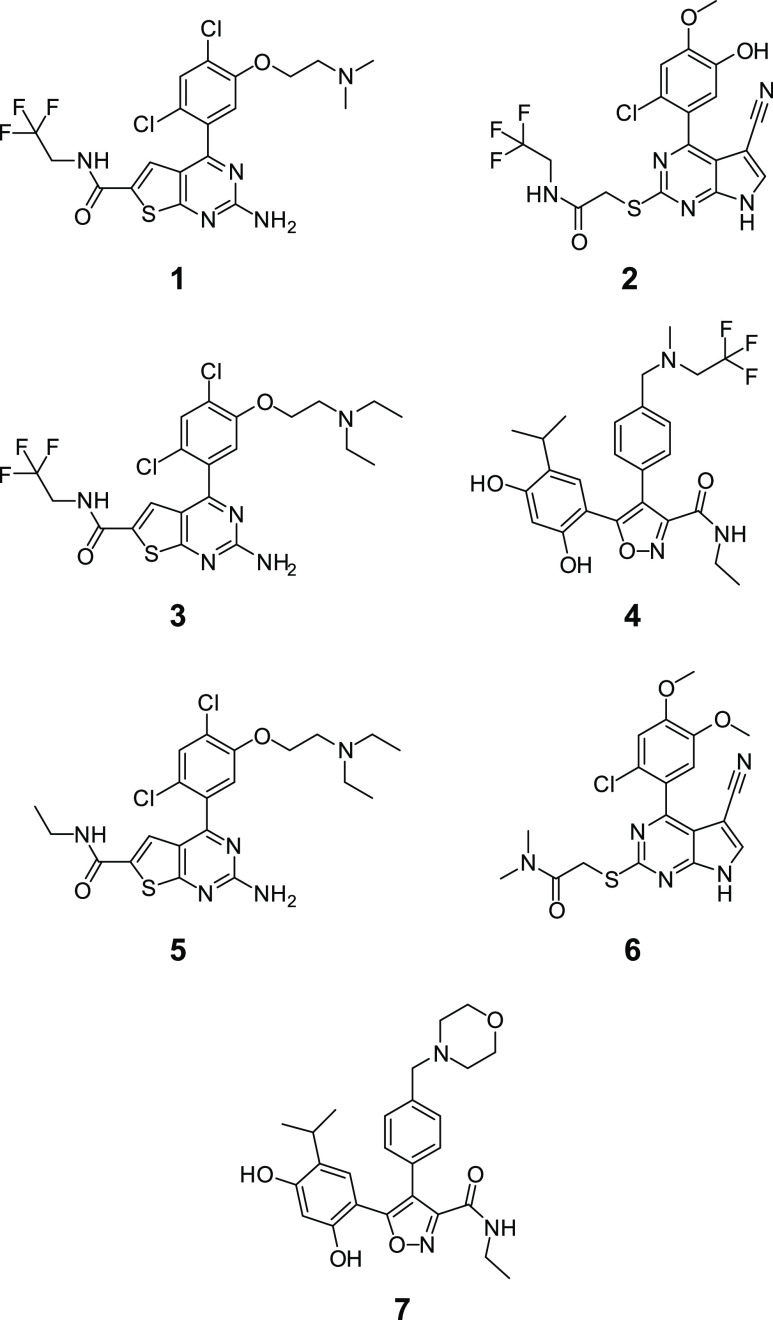
Chemical Structures of the Compounds Analyzed in This Study

**Scheme 1:**
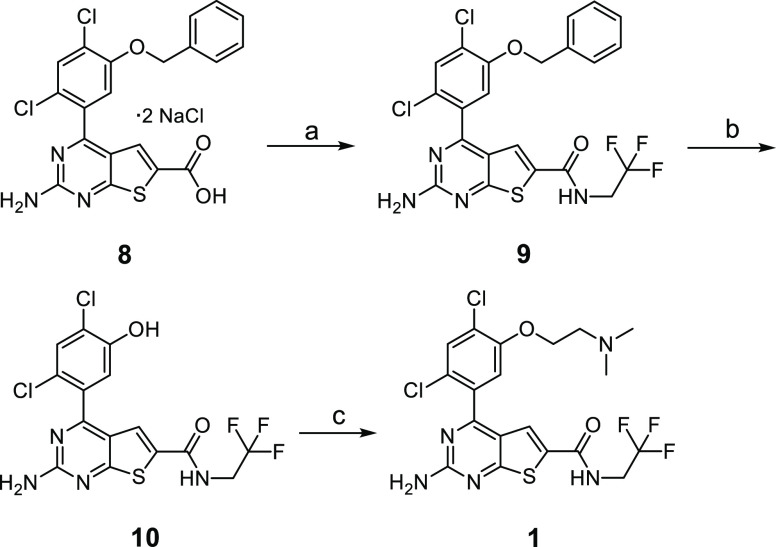
Synthesis of 2-Aminothieno[2,3-*d*]pyrimidine Inhibitor **1** Reagents and conditions: (a) HATU, CF_3_CH_2_NH_2_, DMF, diisopropylamine; 60 °C, 16 h; (b) BCl_3_, DCM, −78 °C to rt; (c) HOCH_2_CH_2_NMe_2_, DIAD, PPh_3_, THF, rt 16 h.

**Scheme 2:**
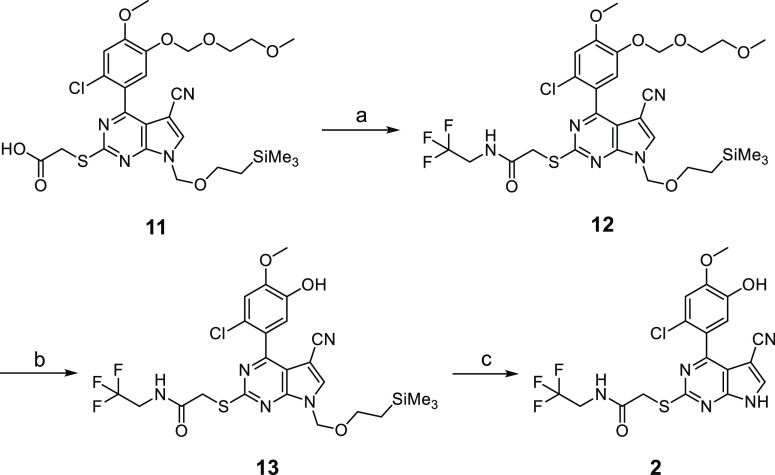
Synthesis of 4-Aryl-5-cyanopyrrolo[2,3-*d*] Pyrimidine Hsp90 Inhibitor **2** Reagents and conditions: (a) CF_3_CH_2_NH_2_, HBTU, MeCN, rt; (b) PPTS, *i*-PrOH, 85 °C, 16 h; (c) TBAF, H_2_N(CH_2_)_2_NH_2_,THF, 40 °C.

**Scheme 3:**
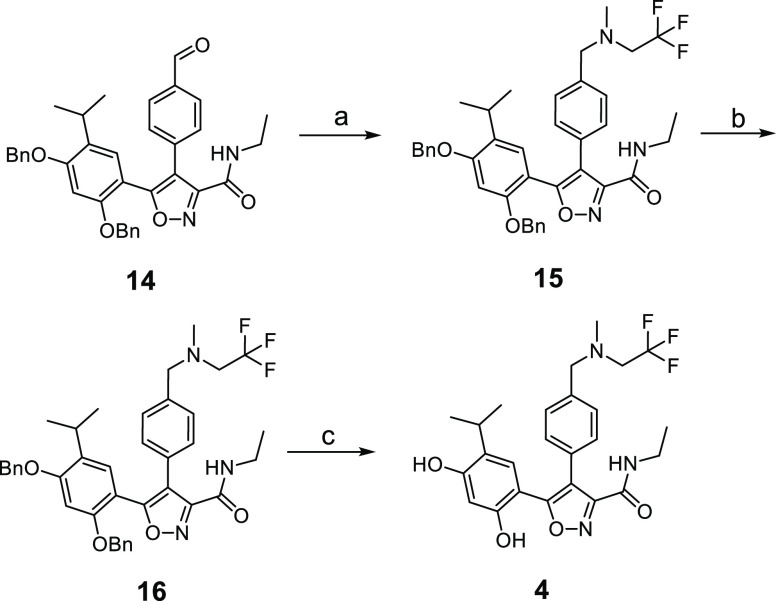
Synthesis of 4,5-Diaryl Isoxazole Hsp90 Inhibitor **4** Reagents and conditions: (a) CF_3_CH_2_NH_2_, Na(OAc)_3_BH, AcOH, MeOH; (b) CF_3_CH_2_NH_2_, CH_2_O, HCO_2_H (c) BCl_3_, DCM, 0 °C.

The test compounds **1–7** used in this study (Chart 1) were selected from three structural chemotypes. Compound **1** is a 2-aminothieno[2,3-*d*]pyrimidine inhibitor and was synthesized in a similar fashion to compounds **3** and **5**, which were previously described,^45^ as shown in Scheme 1. Carboxylic acid **8**(45) was converted to trifluoromethyl amide derivative **9**(45) via a HATU-mediated coupling reaction with 2,2,2-trifluoroethylamine. The phenol moiety was revealed by utilizing boron trichloride in DCM, affording compound **10**.^45^ Subsequent alkylation via the Mitsunobu reaction with *N*,*N*-dimethylethanolamine afforded compound **1**. Compound **2** is an example of the 4-aryl-5-cyanopyrolopyrimidine class of Hsp90 inhibitors and was synthesized by an analogous procedure to compound **6**, which belongs to the same class and was previously described.^46^ The advanced intermediate **11**(46) (Scheme 2) underwent a HBTU-mediated coupling to generate the trifluoro ethyl amide derivative **12**. The MEM-protecting group on the 4-aryl moiety was removed with pyridinium *p*-toluenesulfonate (PPTS) in isopropyl alcohol, affording compound **13**. The SEM-protecting group appended to the pyrrolo nitrogen was subsequently removed with *tert* butyl ammonium fluoride (TBAF) to afford compound **2**. The third compound chemotype studied herein was 4,5-diarylisoxazole, as exemplified by compound **4** and compound **7** (NVP-AUY922).^47^ The synthesis of compound **4** is shown in Scheme 3. The previously described intermediate **14**(47) underwent reductive amination with 2,2,2-trifluoroethylamine to afford amine **15**. *N*-Methylation to **16** and subsequent benzyl protecting group removal with boron trichloride afforded compound **4**. Compound **7** has been previously described, and its synthesis utilizes the same key intermediate (**14**) used for compound **4**.^47^

**Figure 1:**
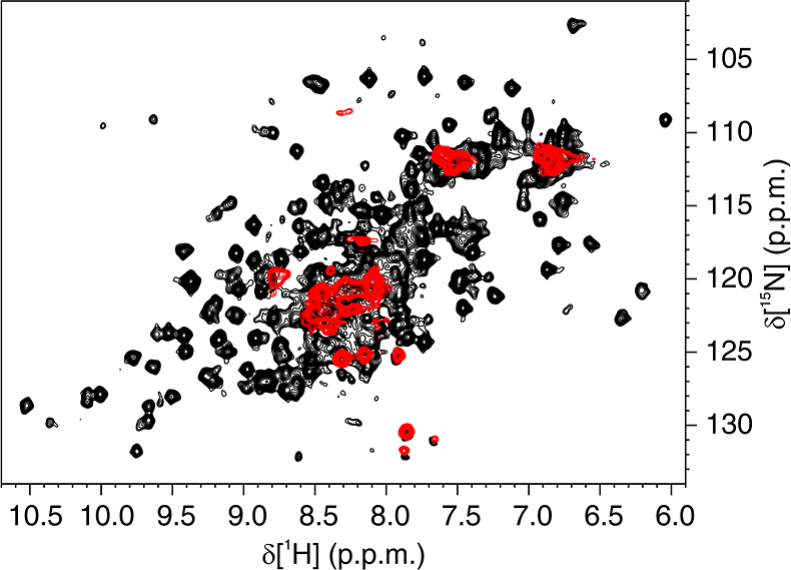
Hsp90_N_ is not detectable by NMR in human cells. Overlay of background-subtracted ^1^H–^15^N SOFAST-HMQC spectra of human cells expressing [U–^15^N]-Hsp90_N_ (red) and the corresponding lysate (black). Only a few amide signals arising from flexible regions of the protein are detected in the in-cell NMR spectra, whereas all the protein signals become visible upon cell lysis.

Hsp90_N_ was investigated in human cells by ^1^H–^15^N in-cell NMR spectroscopy. The domain was overexpressed in HEK293T cells, where it reached an effective concentration of ∼95 μM in the cell pellet and was localized mainly in the cytosol, as observed by SDS-PAGE (Figure S1). The amide signals of [U–^15^N]-labeled protein were not detectable in the ^1^H–^15^N NMR spectra recorded on intact cells, whereas well-resolved amide signals were clearly detected upon cell lysis (Figure 1). The lack of signals from intact cells suggests that the protein interacts diffusely with other cellular components, causing a decrease in the average tumbling rate that results in line broadening beyond detection. These interactions are lost upon cell lysis, making the protein visible in the NMR spectra of the cell lysate. Such behavior is consistent with the biological role of Hsp90α, which can recognize and bind to many different protein substrates.^48^ Hsp90_N_ was therefore taken as a model pharmacological target to which protein-observed in-cell NMR drug screening approaches cannot be easily applied.

**Figure 2:**
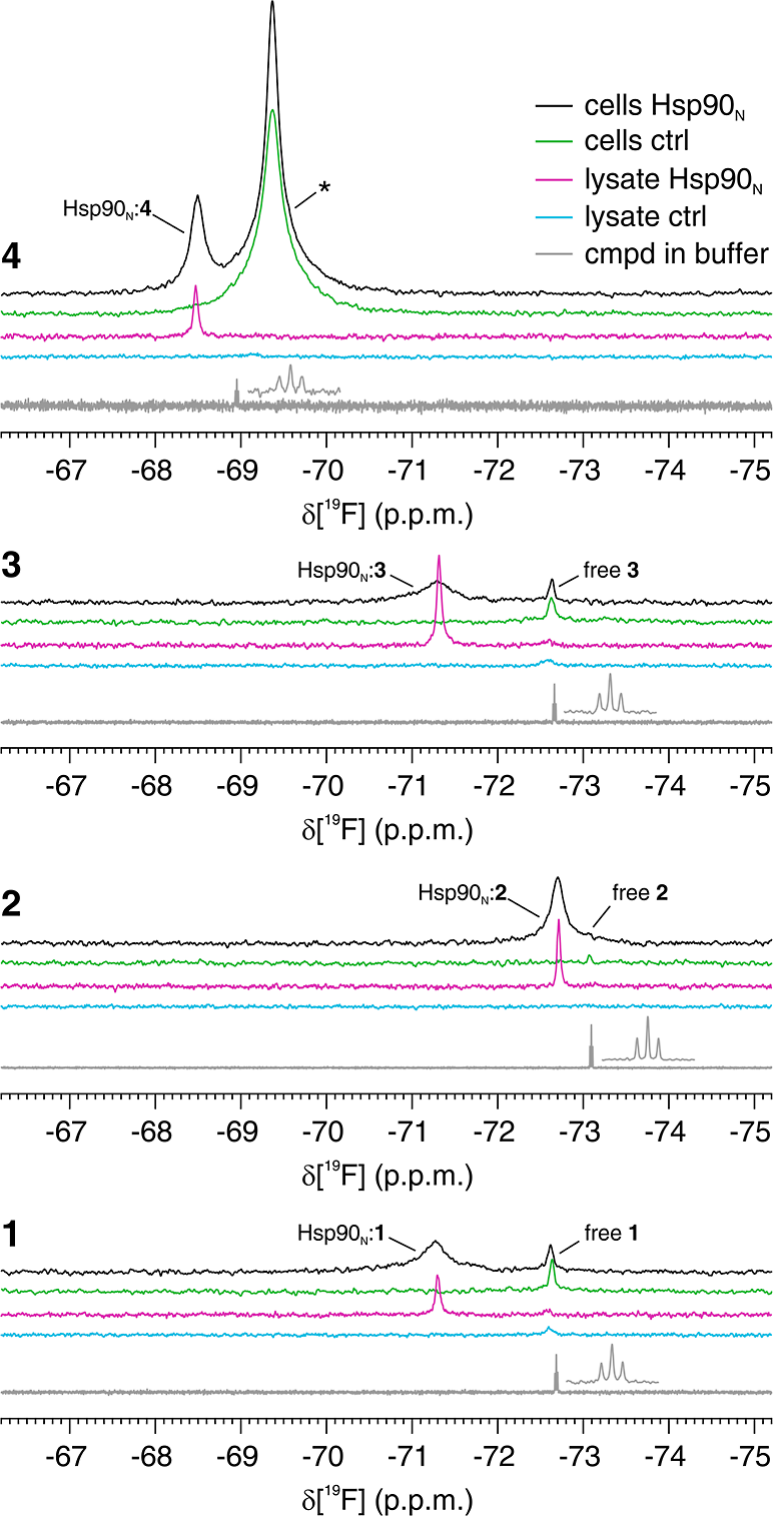
Fluorinated compounds bound to intracellular Hsp90_N_ are detectable by^19^F NMR. In-cell (black, green) and lysate (magenta, cyan) ^19^F NMR spectra from cells expressing Hsp90_N_ (black, magenta) and control cells (green, cyan) treated with compounds **1**–**4**. Reference spectra of the pure compounds are shown in gray. The peaks attributed to the free and Hsp90_N_-bound compounds are labeled accordingly. An additional peak arising from the off-target binding of compound **4** is marked with an asterisk.

**Figure 3:**
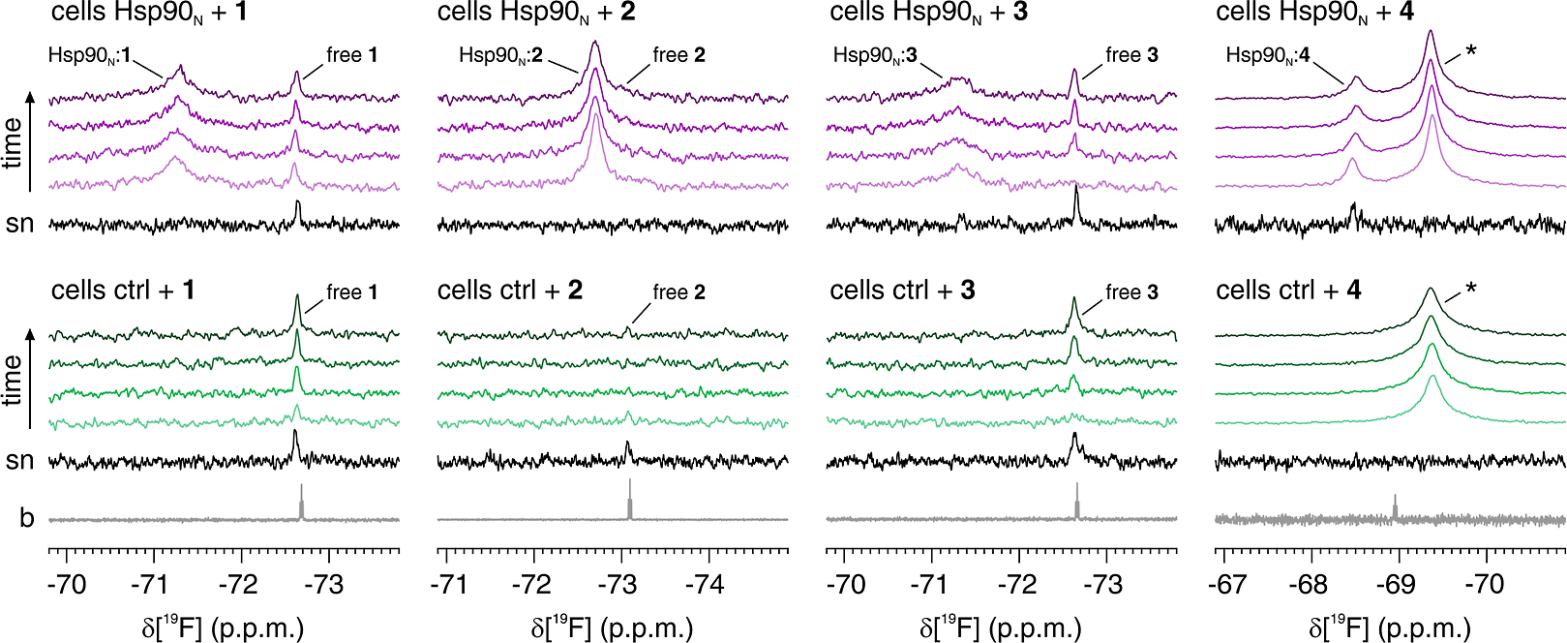
Compounds **1** and **3** are gradually excreted from the cells. Time-resolved in-cell ^19^F NMR spectra recorded over the course of 2 h on cells expressing Hsp90_N_ (violet) and control cells (green) treated with compounds **1**–**4**. Compounds **1** and **3** are excreted from the cells and consequently detected in the supernatant (sn, black). Reference spectra of the pure compounds in buffer (b) are shown in gray. The peaks are labeled as in Figure 2.

To allow the direct observation of ligands by ^19^F NMR as they bind to intracellular Hsp90_N_, compounds containing a trifluoromethyl (CF_3_) group were selected from different series of Hsp90 inhibitors based on their activity against Hsp90_N_ in binding and cell-based growth inhibition assays (Chart 1 and Table 1, compounds **1**–**4**). The compounds were selected from three different chemotypes: aminothieno[2,3-*d*]pyrimidine^45^ (**1**,**3**), 4-aryl-5-cyanopyrrolo[2,3-*d*]pyrimidine^46^ (**2**), and 4,5-diarylisoxazole^47^ (**4**), and they all bind to the ATP binding site of Hsp90_N_, as evidenced by competition binding studies and X-ray crystallography. Compared to other fluorine moieties, the CF_3_ group provides higher sensitivity and more favorable relaxation properties due to its fast rotation. The ^19^F in-cell NMR spectra recorded on cells expressing unlabeled Hsp90_N_ and subsequently treated with the fluorinated compounds contained additional signals in the CF_3_ spectral region which were not present in control cells where Hsp90_N_ was not expressed (Figure 2). These signals were therefore attributed to the protein–ligand complex. This complex was also observed in the ^19^F NMR spectra of the corresponding cell lysates, where they gave rise to sharper signals consistent with faster tumbling in the lysate compared to intact cells (Figure 2). The in-cell detection of the compounds bound to Hsp90_N_ is in stark contrast with the ^1^H–^15^N in-cell NMR analysis, where the same complex did not give rise to observable protein signals, indicating that ligand binding did not affect the interaction of Hsp90_N_ with the cellular environment (Figure S2A). The ^1^H–^15^N spectra of the complex in the corresponding cell lysates were clearly different from those of the unbound protein, further confirming that ligand binding had occurred in the cells (Figure S2B). The signals of free compounds **1** and **3** were also detected in the in-cell ^19^F NMR spectra (Figure 2). Time-resolved in-cell ^19^F NMR experiments revealed that the signal of the free compounds increased over time (Figure 3). The same compounds were also detected in the supernatant recovered after acquisition (Figure 3), indicating that compounds **1** and **3** are gradually excreted in the extracellular medium during the NMR measurement. Conversely, leakage of compound **2** occurred to a much lesser extent and was not observed for compound **4** (Figure 3). Notably, an additional broad ^19^F signal was observed in cells treated with compound **4** (Figure 2). This signal is even stronger than the one arising from the complex with Hsp90_N_ and is also present in the control cells, suggesting that compound **4** interacts with an abundant component natively present in human cells. The lack of a corresponding signal in the cell lysate (Figure 2) suggests that compound **4** binds to cellular membranes or to other nonsoluble components, which are removed from the lysate by centrifugation (see Materials and Methods). The above results indicate that ligand-observed in-cell ^19^F NMR can detect the interaction between fluorinated compounds and an “NMR-invisible” target such as Hsp90_N_ and can also reveal the occurrence of any off-target interaction with abundant cellular components.

**Table 1:** Data on the Compounds Analyzed in This Study

*n*	MW (Da)	IC_50_ (nM)^a^	GI_50_ (nM)^b^	^19^F group
**1**	508.3	282	72	–CF_3_
**2**	471.8	9	46	–CF_3_
**3**	536.4	47	95	–CF_3_
**4**	491.5	53	37^c^	–CF_3_
**5**	482.4	56	73	not present
**6**	431.9	8	34	not present
**7**	465.2	21	16^c^	not present

a IC_50_ from the fluorescence polarization assay.^45^

b From antiproliferative assay in BT474 breast cancer cells.^45,46^

c From antiproliferative assay in HCT116 colon cancer cells.^46,47^

**Figure 4:**
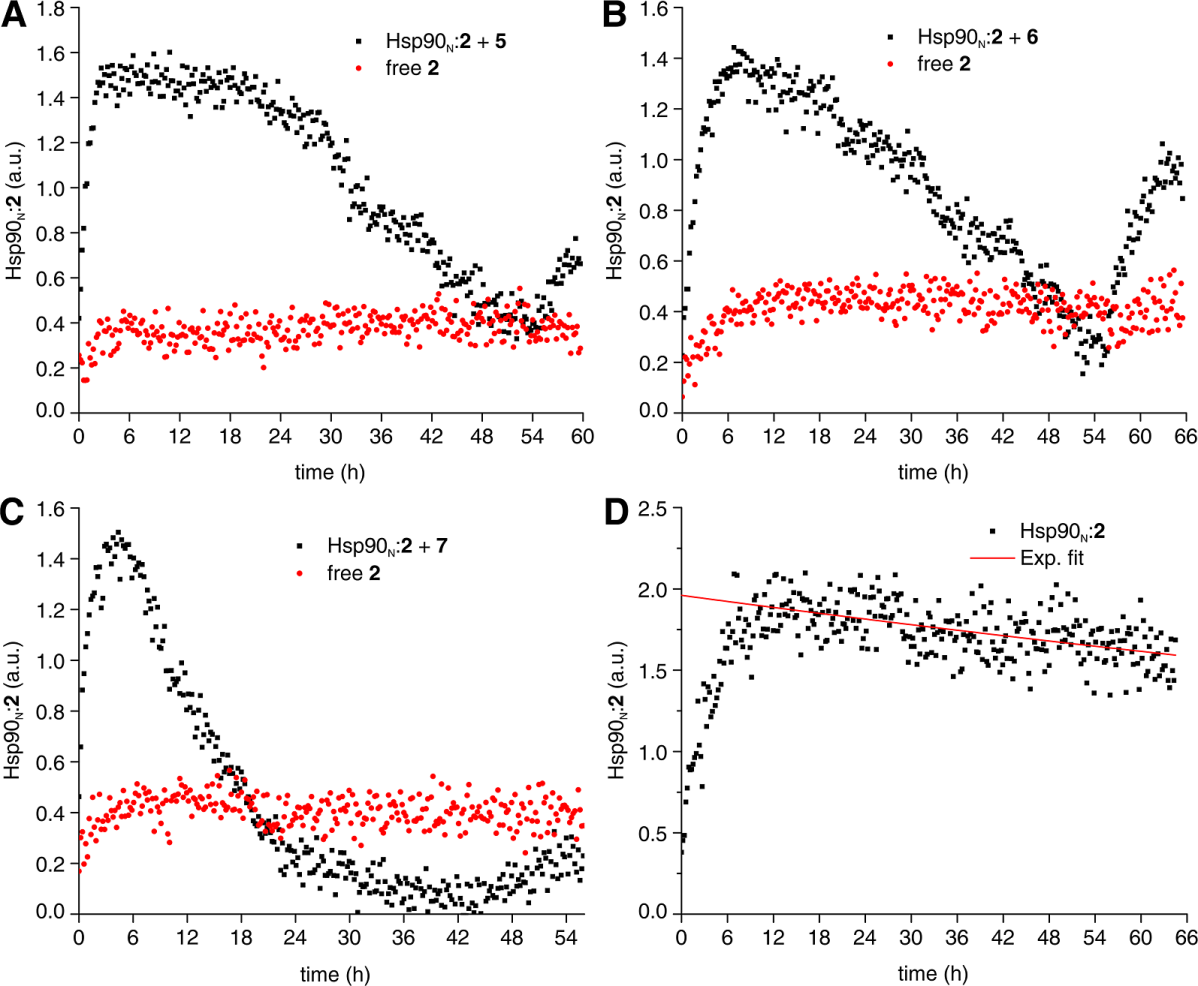
Competition binding by time-resolved in-cell ^19^F NMR. (A–C) In-cell ^19^F NMR competition binding experiments performed in the NMR bioreactor over the course of ∼60 h. The area of the peak from compound **2** in complex with Hsp90_N_ (Hsp90_N_:**2**, black squares) and from extracellular free compound **2** (red circles) is plotted as a function of time. Compound **2** is displaced from Hsp90_N_ at increasing concentrations of test compounds **5** (A), **6** (B), and **7** (C). The concentrations of compound **2** and test compounds at different steps of each bioreactor run are reported in Table S1. (D) Loss of intracellular Hsp90_N_:**2** complex measured as a function of time in a control bioreactor run. The half-life of the complex (215 ± 20 h) was estimated by fitting with an exponential decay (red line).

**Figure 5:**
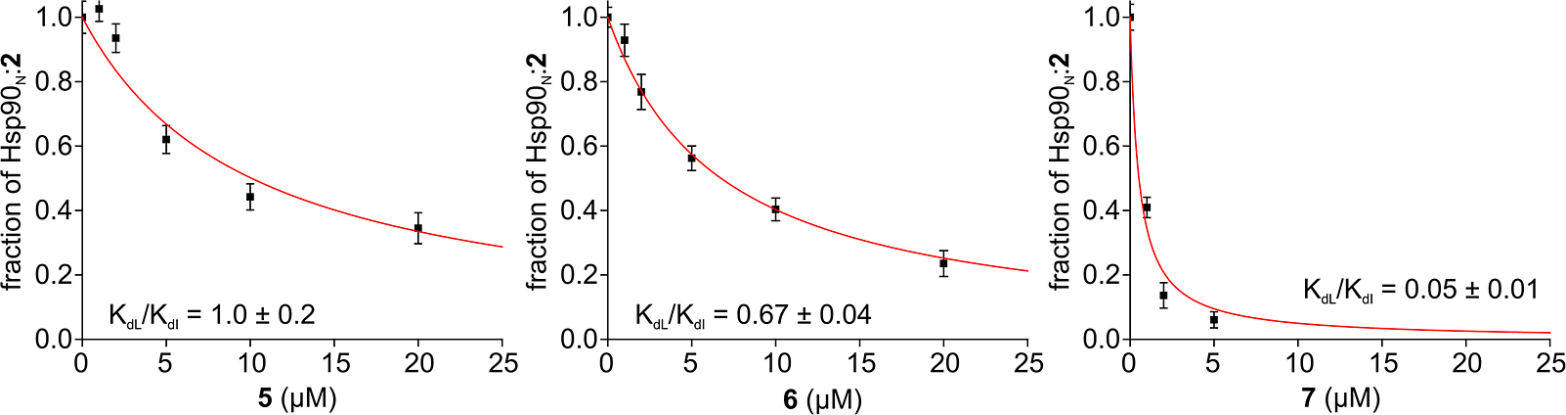
Fitting of the competition binding data. The fraction of the Hsp90_N_:**2** complex reached at the end of each step of the bioreactor runs plotted as a function of test compound concentration. Binding curves from nonlinear curve fitting are shown as red lines. The *K*_dL_/*K*_dI_ obtained for each test compound are reported.

The intracellular binding affinity of three nonfluorinated test compounds (Table 1, compounds **5**–**7**) was then investigated via competition binding by bioreactor-assisted time-resolved in-cell NMR. Compound **5** belongs to the aminothieno[2,3-*d*]pyrimidine^45^ series; compound **6** belongs to the 4-aryl-5-cyanopyrrolo[2,3-*d*]pyrimidine^46^ series. Additionally, we tested compound **7** (NVP-AUY922^47^), which is a high-affinity ligand that also binds at the ATP binding site in the N-terminal domain and was previously studied in Phase I clinical trials for the treatment of cancer. As observed for compounds **1**–**4**, intracellular Hsp90_N_ in complex with compounds **5**–**7** did not give rise to observable signals in the ^1^H–^15^N in-cell NMR spectra, whereas the same complexes were clearly observed in the corresponding cell lysates (Figure S3). Compound **2** was chosen as a spy ligand for the in-cell ^19^F NMR competition binding experiments as it was not excreted from the cells and did not interact with other cellular components. In each experiment, cells expressing Hsp90_N_ were continuously perfused in the bioreactor with fresh medium containing compound **2** at a constant concentration and analyzed by time-resolved ^19^F NMR, while the concentration of the test compound was increased stepwise over the course of the experiment (Figures S4–S6). The displacement of compound **2** upon binding of the test compound to Hsp90_N_ was quantified from the decrease in intensity of the ^19^F signal of the complex (Figure 4A–C). The total amount of intracellular Hsp90_N_ was found to slightly decrease during the course of a control experiment, likely due to the loss of a small number of cells under flow conditions or due to cell rupture (Figure 4D). The signal intensity at each step of the competition binding curves was therefore corrected to compensate for the gradual loss of the target (see Materials and Methods). The fraction of Hsp90_N_ bound to compound **2** as a function of test ligand concentration was then fitted with eq 1 to retrieve the intracellular *K*_dI_/*K*_dL_ ratio for each test ligand (Figure 5 and Table 2). Equation 1 assumes that the free ligand concentration inside and outside of the cells is equal at equilibrium.

**Table 2:** Affinity Constants Derived from Real-Time In-Cell ^19^F NMR Data^d^

compound	IC_50_ (nM)^a^	*K*_dI_/*K*_dI_ by in-cell NMR	*K*_i_ (nM)	*K*_d_ back-calculated from cmpd **6** (nM)
**2** (spy)	9	**1**	n.a.	0.60 ± 0.04
**5**	56	1.0 ± 0.2	42	0.6 ± 0.1
**6**	8	0.67 ± 0.04	<1^b^	**0.4**^c^
**7**	21	0.05 ± 0.01	9	0.03 ± 0.01

a IC_50_ from the fluorescence polarization assay.^45^

b Calculated from FP IC_50_.

c From SPR.^46^

d Reference values in each column are shown in bold.

The obtained affinity ratios ranged from ∼1 to ∼0.05, indicating that all test compounds bind to Hsp90_N_ with higher affinity than the spy ligand. In principle, knowing the intracellular *K*_d_ of a spy ligand would allow calculating the *K*_d_ of each test compound. However, determining the intracellular binding affinities of strong ligands, such as those analyzed here, by direct titration is not straightforward. Furthermore, binding affinities determined in vitro, which could be used as a proxy in the absence of in-cell data, are often dependent on the assay employed and are not easily compared to one another (see Table 1). To estimate the range of binding affinities measurable by our approach, the *K*_d_ of compound **6** previously measured in vitro by surface plasmon resonance (SPR) [*K*_d_(**6**) = 0.4 nM]^46^ was used to back-calculate the intracellular *K*_d_ of the spy ligand [*K*_d_(**2**) = 0.6 nM], from which those of compounds **5** and **7** were calculated. The obtained values ranged from ∼0.6 to ∼0.03 nM (Table 2) indicating that, assuming that the real *K*_d_(**6**) in vivo does not deviate by orders of magnitude from the value reported in vitro, this approach can be applied to measure subnanomolar binding affinities in human cells.

Overall, the results obtained by in-cell ^19^F NMR indicate that the fluorinated compounds (**1**–**4**) investigated in this study permeated the cells within the time frame of the experiment and reached and quantitatively bound intracellular Hsp90_N_. Furthermore, time-resolved competition binding analysis showed that all three nonfluorinated test compounds (**5**–**7**) were able to permeate the cells, engage the intracellular target, and displace the spy ligand (**2**). These results are generally consistent with the IC_50_ and GI_50_ values reported in Table 1, which show that these compounds are strong inhibitors of Hsp90 and readily enter the cells and affect cell proliferation. A direct comparison of the values is not possible due to the fact that the in vitro inhibition depends on the experimental conditions and that cell proliferation assays do not prove direct binding to the intracellular target. Arguably, given that ^19^F NMR spectroscopy provides quantitative measurement of target engagement in the cellular context, our approach may prove to be more reliable for ranking the efficacy of active compounds in the future. Indeed, ranking the compounds **5**–**7** based on the relative *K*_d_s shows that compound **7** is the best candidate for preclinical and clinical tests, and, notably, this result correlates with its reported GI_50_ value but not with the IC_50_ and *K*_i_ values, underscoring the benefit of ranking the relative affinities directly in cells.

## Conclusions

To increase the success of potential drugs in preclinical and clinical trials, it is important to assess their efficacy directly in the cellular environment, in terms of bioavailability, target engagement, and binding affinity, at the early steps of drug development. Typically, *K*_d_ and *K*_i_/IC_50_ are measured on the isolated target, while bioavailability assays are typically aimed at determining the absorption through the intestinal lumen for oral availability using, e.g., Caco-2 model systems.^49^ Target engagement remains more elusive to direct measurement and cannot be inferred from the output of cell-based assays (e.g., cell proliferation), which may give rise to false positives in the case of off-target binding, leading to the wrong mechanism of action. Several approaches exist for target engagement which require the modification of the compound to make it suitable for fluorescence, bioluminescence, or affinity-based methods coupled with mass spectrometry.^50^ More recently, cellular thermal shift assay (CETSA) approaches have been employed to detect target engagement with high throughput and without requiring chemical modification of the compounds.^50,51^

^19^F NMR is an established and effective approach to investigating ligand–target interactions in vitro and has been recently applied to study both small and large molecules directly in living cells. Here, we have shown that ligand-observed in-cell ^19^F NMR can reveal target engagement and provide quantitative information on the binding affinity of strong ligands toward their target, the N-terminal domain of Hsp90α, inside intact human cells. The in-cell ^19^F NMR approach reported here leverages the main advantages of the ^19^F nucleus, i.e., the high gyromagnetic ratio, the high chemical sensitivity, and the absence of background in biological systems. Once a suitable fluorinated compound is chosen as a spy ligand, the relative intracellular affinity of nonfluorinated compounds can be determined by competition binding. Importantly, the favorable relaxation properties of the CF_3_ group made it possible to observe the NMR signal of a fluorinated ligand involved in a stable complex with its target, even when the latter could not be detected by ^1^H NMR due to interactions with the cellular environment. As long as the CF_3_-containing group retains fast internal motions when the spy ligand is bound to the target, this approach should be applicable to other pharmacologically relevant targets that cannot be directly observed by NMR due to their large size and/or intracellular interactions, and it only requires one appropriately chosen fluorinated compound to enable the screening of nonfluorinated molecules. The range of affinities measured by competition binding depends on the *K*_d_ of the spy ligand. Here, a spy ligand binding to Hsp90_N_ with subnanomolar affinity was chosen, which allowed measuring *K*_d_s as low as ∼50 pM. In cases where the *K*_d_ of the spy ligand is not known, the approach can still be used to rank the tested ligands by their relative affinity. In principle, lower-affinity ligands (e.g., with *K*_d_s in the range of micro-to-nanomolar) can be measured by choosing a spy ligand with a higher *K*_d_. However, molecules binding with *K*_d_s in the high-micromolar and millimolar ranges will likely experience intermediate-to-fast exchange, making this approach unsuitable for screening fragments or other weak binders.

In the context of drug development, the insight obtained by in-cell ^19^F NMR will be especially beneficial at the stage of lead compound optimization, when relatively few molecules need to be extensively studied in terms of bioavailability and target engagement to rule out potential pitfalls in the preclinical and clinical trials. Compared to established assays such as CETSA, widespread application of in-cell ^19^F NMR is limited by its lower throughput due to the intrinsically low sensitivity of NMR spectroscopy: the intracellular target needs to be overexpressed to ∼10 μM or higher in order to allow the detection of the complex. On the other hand, our approach preserves cell viability and metabolic activity and therefore, in principle, allows simultaneous real-time measurement of different cellular parameters (e.g., metabolic and lipid composition, ATP production, and enzymatic activity). In principle, additional information on the local dynamics and exchange rates of the complex may be obtained by line shape and chemical shift perturbation analysis,^52^ and more in-depth analysis of the time-resolved displacement curves may allow untangling the uptake kinetics of the inbound drug and the off-rate constant of the outbound drug, providing valuable insights on drug binding kinetics.^53^ We believe that the approach presented here will be especially powerful when applied to challenging drug targets such as highly dynamic domains for which binding affinities might change dramatically with the in-cell target conformation for intrinsically disordered regions forming high-affinity fuzzy complexes and for developing protein–protein interactions inhibitors. Drugs interacting with these target categories cannot be easily assayed with classical methods and are currently greatly underrepresented. Overall, we expect that in-cell ^19^F NMR will prove useful to increase the success rate of lead compounds toward both classical and more challenging targets once they move on to the next steps of drug development.

## Experimental Section

### General Procedures

^1^H (400 MHz) and ^13^C (100.6 MHz) NMR analyses were carried out on a Bruker DPX-400 MHz spectrometer. ^1^H NMR spectra were also recorded at 250 MHz on a Bruker AC250 and at 500 MHz on a Bruker 500 MHz Ultrashield spectrometer. The chemical shift of the sample solvent was used as a spectral reference. The ^1^H NMR data is reported, indicating the chemical shift (δ) as parts per million (ppm), the multiplicity (s, singlet; d, doublet; t, triplet; q, quartet; sept, septet; m, multiplet; br, broad; dd, doublet of doublets, etc.), the integration (e.g., ^1^H), and the coupling constant (*J*) in Hertz (Hz) (app, apparent coupling on broadened signals). ^13^C NMR data is reported indicating the chemical shift (δ) as parts per million (ppm) and, in some cases, annotated with the carbon multiplicity: (CH3) for primary carbon, (CH2) for secondary carbon, (CH) for tertiary carbon, and (C) for quaternary carbon. Deuterated solvents were purchased from either Sigma–Aldrich or Fluorochem.

LC–MS analyses were performed on an HP1100 instrument (*method A*) with a Luna 3 DM, C18(2), 30 mm × 4.6 mm i.d. column from Phenomenex at a temperature of 22 °C at a flow rate of 2 mL/min. Solvents were purchased from ROMIL Ltd., Waterbeach, UK. The following solvent systems were used: solvent A, HPLC-grade water + 10 mM ammonium acetate + 0.08% v/v formic acid. Solvent B, 95% v/v HPLC-grade acetonitrile + 5% v/v solvent A + 0.08% v/v formic acid. Gradient: 95:5 solvent A/solvent B, 0.00–0.25 min; 95:5–5:95 solvent A/solvent B, 0.25–2.50 min; 5:95 solvent A/solvent B, 2.50–3.75 min. UV detection was at 230, 254, and 270 nm. The mass spectrometer was an HP1100MSD, Series A instrument, operating in the positive or negative ion electrospray ionization mode. The molecular weight scan range was 120–1000. Samples were supplied as a 1 mM solution in DMSO with a 5 DL partial loop fill injection. LC purities were assigned one of three values: 85–90, 90–95, or >95%. Chemical samples were also analyzed by a separate LC–MS system (*method B*) using a Micromass LCT/Water’s Alliance 2795 HPLC system with a Discovery 5 Dm, C18, 50 mm × 4.6 mm i.d. column from Supelco at a temperature of 22 °C and a flow rate of 1 mL/min. The following solvent systems were used: solvent A, MeOH. Solvent B, 0.1% Formic acid in water. Gradient starting with 10% A: 90% B from 0 to 0.5 min, then 10% A: 90% B to 90% A: 10% B from 0.5 to 6.5 min, and continuing at 90% A: 10% B up to 10 min. From 10 to 10.5 min, the gradient reverted back to 10% A: 90% B, where the concentrations remained until 15 min. UV absorption was measured at 254 nm; ionization was positive or negative ion electrospray. The molecular weight scan range was 50–1000. Samples were supplied as 1 mg/mL in DMSO or MeOH with 3 μL injected on a partial loop fill. All compounds used in this study were >95% pure by HPLC analysis.

#### 2-Amino-4-[2,4-dichloro-5-(2-dimethylamino-ethoxy)-phenyl]-thieno[2,3-*d*]pyrimidine-6-carboxylic Acid (2,2,2-Trifluoro-ethyl) Amide (**1**)

Compound **10**(45) (100 mg, 0.23 mmol) was dissolved in anhydrous THF (30 mL). *N*,*N*-Dimethylethanolamine (27 mL, 0.27 mmol) was added, followed by triphenylphosphine (90 mg, 0.27 mmol) and diisopropyl azodicarboxylate (67 mL, 3.29 mmol). The reaction mixture was stirred at rt for 16 h, then EtOAc (25 mL) was added, and the mixture was washed sequentially with water (2 × 20 mL) and sat. NaCl solution (20 mL). The organic phase was dried over Na_2_SO_4_, filtered, and the filtrate solvents removed in vacuo to afford a yellow oil, which was purified by flash chromatography on silica gel (25 g) eluting with 10% MeOH in DCM to afford the title compound **1** (58 g, 49%) as a colorless powder: LC–MS *t*_R_ = 0.889 min; *m*/*z* = 510, 508 [M + H]^+^; ^1^H NMR (400 MHz, DMSO-*d*_6_): δ 2.23 (s, 6H), 2.68 (t, *J* = 5.6 Hz, 2H), 3.98–4.13 (m, 2H), 4.18 (t, *J* = 5.6 Hz, 2H), 7.36 (s, 2H), 7.42 (s, 1H), 7.76 (s, 1H), 7.84 (s, 1H), 9.17 (t, *J* = 6.3 Hz, 1H); ^19^F NMR (376 MHz, DMSO-*d*_6_): δ −70.49; ^13^C NMR (100.6 MHz, DMSO-*d*_6_): δ 40.0 (CH2), 45.6 (CH3), 57.3 (CH2), 67.9 (CH2), 115.3 (CH), 121.1 (C), 122.3 (C), 123.1 (C), 123.1 (CH), 124.6 (C), 130.4 (CH), 130.4 (C), 135.5 (C), 152.9 (C), 161.3 (C), 161.8 (C), 162.0 (C), 171.4 (C); HRMS, calcd for C_19_H_19_Cl_2_F_3_N_5_O_2_S [M + H]^+^, 508.0589; found, 508.0583; HPLC (*method A*) 99.8% (*t*_R_ = 0.889 min).

#### 2-[4-(2-Chloro-4-methoxy-5-[(2-methoxyethoxy)methoxy]-phenyl)-5-cyano-7-(2-trimethylsilanyl-ethoxymethyl)-7*H*-pyrrolo[2,3-*d*]pyrimidin-2-ylsulfanyl]-*N*-(2,2,2-trifluoroethyl)acetamide (**12**)

To a solution of 2-[4-(2-chloro-4-methoxy-5-[(2-methoxyethoxy)methoxy]-phenyl)-5-cyano-7-(2-trimethylsilanyl-ethoxymethyl)-7*H*-pyrrolo[2,3-*d*]pyrimidin-2-ylsulfanyl]-acetic acid **11**,^54^ (450 mg, 0.740 mmol) in anhydrous MeCN (10 mL) were added sequentially triethylamine (0.309 mL, 2.22 mmol), 2,2,2 trifluoroetan-1-amine hydrochloride (150 mg, 1.11 mmol), and HBTU (*O*-(benzotriazol-1-yl)-*N*,*N*,*N*′,*N*′-tetramethyluronium hexafluorophosphate, 421 mg, 1.11 mmol). After 1 h of stirring at ambient temperature, the reaction mixture was partitioned between EtOAc (50 mL) and aqueous NH_4_Cl solution (50 mL). The combined organics were dried (Na_2_SO_4_) and evaporated in vacuo. The resultant crude oil was purified by flash chromatography on SiO_2_ (70 g) eluting with hexane to 50% EtOAc/hexane to afford the title compound **12** (474 mg, 93%) as a colorless solid: LC–MS (*method B*) *t*_R_ = 3.78; *m*/*z* = 609 [M + H]^+^; ^1^H NMR (400 MHz, DMSO-*d*_6_): δ −0.07 (s, 9H), 0.80–0.87 (m, 2H), 3.17 (s, 3H), 3.42–3.47 (m, 2H), 3.56–3.63 (m, 2H), 3.72–3.77 (m, 2H), 3.89 (s, 3H), 3.86–3.97 (m, 2H), 4.04 (s, 2H), 5.26 (s, 2H), 5.64 (s, 2H), 7.26 (s, 1H), 7.27 (s, 1H), 8.64 (s, 1H), 8.80 (t, *J* = 6.3 Hz, 1H).

#### 2-[4-(2-Chloro-5-hydroxy-4-methoxy-phenyl)-5-cyano-7-(2-trimethylsilanyl-ethoxymethyl)-7*H*-pyrrolo[2,3-*d*]pyrimidin-2-ylsulfanyl]-*N*-(2,2,2-trifluoroethyl)acetamide (**13**)

To a solution of compound **12** (474 mg, 0.688 mmol) in ^*i*^PrOH (10 mL), PPTS (190 mg, 0.757 mmol) was added, and the mixture was heated at 85 °C under an N_2_ atmosphere overnight. The reaction mixture was allowed to cool and partitioned between EtOAc (2 × 20 mL) and NH_4_Cl solution (20 mL). The phases were separated and organic phase-dried over anydrous Na_2_SO_4_; the organics were filtered and filtrate evaporated in vacuo to give a brown oil. This crude material was purified by flash chromatography on silica gel (25 g) eluting with a gradient of 33–50% EtOAc in Heptane to afford the title compound **13** (310 mg, 75%) as a white foam: LC–MS (*method B*) *t*_R_ = 3.62 min; *m*/*z* = 602 [M + H]^+^; ^1^H NMR (400 MHz, DMSO-*d*_6_): δ −0.08 (s, 9H), 0.79–0.86 (m, 2H), 3.55–3.62 (m, 2H), 3.87 (s, 3H), 3.87–3.98 (m, 2H), 4.03 (s, 2H), 5.63 (s, 2H), 6.89 (s, 1H), 7.14 (s, 1H), 8.62 (s, 1H), 8.81 (t, *J* = 6.3 Hz, 1H), 9.62 (s, 1H).

#### 2-[4-(2-Chloro-5-hydroxy-4-methoxy-phenyl)-5-cyano-7*H*-pyrrolo[2,3-*d*]pyrimidin-2-ylsulfanyl]-*N*-(2,2,2-trifluoroethyl)acetamide (**2**)

To a solution of compound **13** (80 mg, 0.133 mmol) in anhydrous THF (3 mL) was added ethylenediamine (0.027 mL, 0.399 mmol) and 1.0 M TBAF in THF solution (0.798 mL, 0.798 mmol). The mixture was heated at 40 °C overnight. The reaction mixture was then allowed to cool to ambient temperature and was then partitioned between EtOAc (30 mL) and water (30 mL). The organic layer was dried (Na_2_SO_4_), filtered, and the filtrates were evaporated in vacuo to give a crude oil (ca. 65 mg). This crude material was purified by flash chromatography on silica gel (10 g) eluting with a gradient of 0–1% methanol in DCM to afford the title compound **2** (51 mg, 81%) as a colorless solid: LC–MS (*method A*) *t*_R_ = 0.875 min; *m*/*z* = 472 [M + H]^+^; ^1^H NMR (400 MHz, CD_3_OD): δ 3.90 (q, 2H, *J* = 9.4 Hz), 3.94 (s, 3H), 4.01 (s, 2H), 6.91 (s, 1H), 7.10 (s, 1H), 8.11 (s, 1H); ^13^C NMR (100.6 MHz, CD_3_OD): δ 35.5 (CH2), 41.7 (CH2), 56.8 (CH3), 86.9 (C), 113.8 (CH), 114.5 (C), 115 (C), 118.1 (CH), 124.1 (C), 125.7 (C), 128.3 (C), 137.1 (CH), 146.9 (C), 151.2 (C), 153.9 (C), 159.6 (C), 165.8 (C), 172.1 (C); HRMS, calcd for C_18_H_14_ClF_3_N_5_O_3_S [M + H]^+^ found, 472.0452 requires 472.0458; HPLC 99.4% (*t*_R_ = 0.875 min).

#### 5-[2,4-Bis(benzyloxy)-5-(propan-2-yl)phenyl]-*N*-ethyl-4-(4-{[(2,2,2-trifluoroethyl)amino]methyl}phenyl)-1,2-oxazole-3-carboxamide (**15**)

Compound **14**(47) (575 mg, 1.0 mmol) was added to a solution of 2,2,2-trifuoroethylamine (500 mg, 5 mmol) in DCM (20 mL). Sodium triacetoxyborohydride (1.5 g, 7 mmol) was added, and the resulting suspension was stirred at ambient temperatures overnight. The reaction mixture was diluted with DCM (30 mL), and the mixture was washed sequentially with aqueous sodium hydroxide (1.0 M, 20 mL), water (2 × 20 mL), and sat. aqueous NaCl solution. The organic phase was dried over anhydrous magnesium sulfate, filtered, and filtrate evaporated to afford the title compound (**15**) (708 mg, quant.) as a brown gum used directly without further purification.

#### 5-[2,4-Bis(benzyloxy)-5-(propan-2-yl)phenyl]-*N*-ethyl-4-(4-{[methyl(2,2,2-trifluoroethyl)amino]methyl}phenyl)-1,2-oxazole-3-carboxamide (**16**)

A solution of formaldehyde (35 wt % aq, 2.5 mL) was added to a mix of compound **15** (700 mg, 1.0 mmol) and formic acid, and the suspension was heated at 100 °C for 18 h. The resulting solution was allowed to cool to ambient temperature, DCM (50 mL) was added, and the organic phase was washed sequentially with aqueous ammonia solution (35% w/w, 25 mL), water (2 × 25 mL), and sat. aqueous NaCl solution (25 mL). The organic phase was dried over anhydrous magnesium sulfate, filtered, and evaporated to afford yellow gum, which was purified by flash chromatography on silica gel (70 g) eluting with ethyl acetate:hexane (1:3) to afford the title compound (**16**) as an off-white solid: TLC *R*_f_ = 0.12 (EtOAc/hexane 1:3); ^1^H NMR (400 MHz, CDCl_3_): δ 1.04 (d, *J* = 6.9 Hz, 6H), 1.22 (t, *J* = 7.3 Hz, 3H), 2.42 (s, 3H), 3.04 (q, JHF = 9.5 Hz, 2H), 3.15–3.28 (m, 1H), 3.40–3.49 (m, 2H), 3.70 (s, 2H), 4.82 (s, 2H), 4.98 (s, 2H), 6.46 (s, 1H), 6.74 (t, *J* = 5.8 Hz, 1H), 7.07 (s, 1H), 7.14–7.42 (m, 14H).

#### 5-[2,4-Dihydroxy-5-(propan-2-yl)phenyl]-*N*-ethyl-4-(4-{[methyl(2,2,2-trifluoroethyl)amino]methyl}phenyl)-1,2-oxazole-3-carboxamide (**4**)

Boron trichloride in DCM solution (1.0 M, 2.5 mL, 2.5 mmol) was added dropwise to a solution of compound **16** (275 mg, 0.41 mmol) in DCM (5 mL) under a nitrogen atmosphere and cooled with a dry ice/acetone bath (ca. −78 °C). The resulting suspension was stirred for 30 min. The cooling bath was removed, and the mixture was stirred for 2 h at ambient temperature. The reaction mixture was cooled with an ice-water bath, methanol (5 mL) was added dropwise, and the resulting solution was stirred for 10 min at ambient temperature. Then, solvents were removed in vacuo to as a brown gum, which was purified by PREP HPLC (methods within Supporting Information) to afford title compound **4** (90 mg, 43%) as a colorless solid: LC–MS *t*_R_ = 1.217 min, *m*/*z* = 492 [M + H]^+^; ^1^H NMR (400 MHz, DMSO-*d*_6_): δ 0.92 (d, *J* = 6.9 Hz, 6H), 1.07 (t, *J* = 7.2 Hz, 3H), 2.30 (s, 3H), 2.97 (hep, *J* = 6.9 Hz, 1H), 3.17–3.29 (m, 4H), 3.68 (s, 2H), 6.44 (s, 1H), 6.74 (s, 1H), 7.17–7.28 (m, 4H), 8.82 (t, *J* = 5.7 Hz, 1H), 9.64 (s, 1H), 9.75 (s, 1H); ^19^F NMR (376 MHz, DMSO-*d*_6_): δ −67.94; ^13^C NMR (100.6 MHz, DMSO-*d*_6_): δ 14.3 (CH3), 22.3 (CH3), 25.4 (CH), 33.7 (CH2), 42.2 (CH3), 56.0 (CH2), 61.0 (CH2), 102.7 (CH), 104.5 (C), 114.6 (C), 125.6 (C), 126.2 (C), 127.7 (CH), 128.2 (CH), 128.6 (C), 128.9 (CH), 137.3 (C), 154.7 (C), 157.4 (C), 157.8 (C), 159.8 (C), 166.2 (C); HRMS, calcd for C_25_H_29_F_3_N_3_O_4_ [M + H]^+^, 492.2110; found, 492.2107; HPLC 100% (*t*_R_ = 1.217 min).

### Gene Cloning

To generate the mammalian expression plasmid, the cDNA encoding the N-terminal ATP-binding domain of Hsp90α (henceforth Hsp90_N_, amino acids 9-236, GenBank: NP_005339.3) was amplified by PCR and cloned into the pHLsec vector^54^ between *Eco*RI and XhoI restriction sites, following a previously reported cloning strategy.^55^ A Kozak sequence was inserted downstream of the *Eco*RI site, while a stop codon was inserted upstream of the XhoI site. Cloning between the above restriction sites results in the removal of a N-terminal signal peptide and a C-terminal histidine tag, which were present in the original vector. The resulting expression vector (pHL-Hsp90_N_) encodes the native protein sequence for cytoplasmic expression. The clone was verified by DNA sequencing.

### Human Cell Culture and Transfection

HEK293T cells (ATCC CRL-3216) were seeded in uncoated 75 cm^2^ plastic flasks and grown in Dulbecco-modified Eagle medium (DMEM) with high glucose (Gibco) supplemented with l-glutamine, penicillin, streptomycin, and 10% fetal bovine serum (FBS, Gibco) at 37 °C with 5% CO_2_ in a humidified atmosphere. The cells were transiently transfected with the pHL-Hsp90_N_ plasmid using polyethylenimine (PEI), with a DNA/PEI ratio of 1:2 (25 μg DNA, 50 μg PEI), following a reported protocol.^55^ Commercial DMEM medium was used for unlabeled in-cell NMR samples; [U–^15^N]-BioExpress 6000 medium (Cambridge Isotope Laboratories) was used for uniformly ^15^N-labeled in-cell NMR samples. Both expression media were supplemented with 2% FBS and antibiotics; the unlabeled medium was also supplemented with l-glutamine.

### Protein Quantification and Localization

The protein expression level was determined by Coomassie-stained SDS-PAGE. Densitometry analysis was performed with ImageJ software.^56^ Lysates from cell samples were run at increasing dilutions together with purified carbonic anhydrase II (obtained as described previously^12^), which has a similar molecular weight, as a reference. The value reported in the main text reflects the protein concentration calculated from cells lysed in one cell pellet volume, therefore corresponding to the effective concentration in the in-cell NMR samples. Protein intracellular localization was assessed by cellular fractionation and analyzed by Coomassie-stained SDS-PAGE. The nuclear, cytosolic, and mitochondrial fractions were obtained using a mitochondria isolation kit for cultured cells (Thermo Scientific), as previously described.^57^ Cells from two 75 cm^2^ flasks were ruptured with a Dounce homogenizer and gently spun. The nuclear fraction was obtained by washing twice the pellet and resuspending it in 1 mL of PBS buffer. The cytosolic and mitochondrial fractions were obtained according to the kit protocol. The mitochondrial fraction was resuspended in 100 μL of PBS buffer.

### Preparation of Cell and Lysate NMR Samples

Cells expressing Hsp90_N_ were detached with trypsin 48 h post-transfection, suspended in DMEM + 10% FBS, washed once with PBS, and resuspended in one cell pellet volume of NMR medium (DMEM supplemented with 90 mM glucose, 70 mM HEPES, and 20% D_2_O). The cell suspension was transferred to a 3 mm Shigemi NMR tube and gently spun to form a soft pellet at the bottom of the tube. After the NMR experiments, the cells were collected, and the supernatant was checked to exclude protein leakage by NMR (Figure S7). Cell lysates were prepared from each cell sample by 8–10 freeze–thaw cycles in 150 μL of PBS buffer, followed by centrifugation at 16,000*g*, 4 °C for 1 h to remove the insoluble fraction. The supernatants were supplemented with 10% D_2_O and placed in a standard 3 mm tube for NMR analysis.

### Cell Encapsulation in Agarose Threads

Cells were encapsulated in agarose threads, as previously reported.^58^ Low-gelling agarose (Sigma–Aldrich) was dissolved at 1.5% (w/v) in PBS at 85 °C, sterilized by filtration, and stored at 4 °C. For cell encapsulation, one aliquot of agarose was melted at 85 °C and kept in solution at 37 °C. Cells collected from one 75 cm^2^ flask were prewarmed at 37 °C and resuspended in 450 μL of agarose solution. The cell–agarose suspension was aspirated into a chromatography PEEK tubing (inner diameter, i.d., 0.75 mm) connected to a 1 mL syringe and cooled down at room temperature for 2 min. Agarose threads were cast into the flow unit NMR tube containing a ∼5 mm-high bottom plug of 1.5% agarose gel and prefilled with 100 μL PBS.

### NMR Bioreactor Setup and Operation

The NMR bioreactor was set up, as previously reported.^58^ Briefly, the 5 mm glass sample tube containing the encapsulated cells was watertight-connected to the tube holder of the bioreactor flow unit (InsightMR, Bruker). A PEEK capillary inlet (i.d. 0.5 mm) was inserted in the sample tube down to ∼6 mm from the bottom, while a polytetrafluoroethylene (PTFE) capillary outlet (i.d. 0.5 mm) was attached at the top of the tube holder. The tubing was temperature-controlled at 37 °C via a circulating water bath (Julabo). The inlet and outlet were connected through PEEK tubing through a four-way valve to a 3-channel peristaltic pump (Reglo ICC, Ismatech) for controlling the medium flow and to a waste container. The flow from two channels of the peristaltic pump was combined using a Y-junction upstream of the valve. During the in-cell NMR experiments, unlabeled DMEM (Sigma-Aldrich D5648, powder, reconstituted in sterile-filtered Milli-Q H_2_O and supplemented with 2% FBS, 10 mM NaHCO_3_, antibiotics, the desired compounds (compounds **1**–**7**) at the chosen concentration, and 2% D_2_O, pH 7.4) was supplied by the two channels of the peristaltic pump at variable flow rates, which were controlled by the pump software. The total flow was kept constant at 0.1 mL/min. Compound concentrations and flow rates at each step of the bioreactor runs are reported in Table S1. The media were contained in 250 or 500 mL reservoir glass bottles kept at 37 °C in the water bath.

### NMR Experiments

^1^H and ^1^H–^15^N NMR spectra on cells expressing U–^15^N-labeled Hsp90_N_ were recorded at 310 K at a 900 MHz Bruker Avance NEO equipped with a 5 mm TCI Cryoprobe. 2D ^1^H–^15^N SOFAST-HMQC spectra (Bruker pulse sequence sfhmqcf3gpph)^59^ were recorded on cell samples and on the corresponding lysates with frequency offsets of 4.7 ppm (^1^H) and 118 ppm (^15^N), spectral windows of 16 ppm (^1^H) and 50 ppm (^15^N), acquisition times of 52.2 ms (^1^H) and 14 ms (^15^N), and an interscan delay of 0.3 s using the shaped pulses Pc9_4_90.1000 and Reburp.1000 for selective ^1^H inversion and refocusing, respectively. The excitation width and offset were set to 6 and 8.7 ppm, respectively. Shaped pulse lengths and power levels were automatically calculated (-DCALC_SP option in the pulse sequence). 64 initial scans and 128 increments were employed, resulting in a total experimental time of 58 min. To remove the background signals arising from the incorporation of ^15^N in other cellular components, each 2D NMR spectrum recorded on cells and lysates was further processed in Topspin (Bruker) by subtracting a spectrum recorded using identical parameters on cells transfected with an empty vector and on the corresponding lysate, as previously described.^55^

^19^F NMR spectra were recorded at 310 K at a 600 MHz Bruker Avance III equipped with a room-temperature SEL-HP probe tuned at 564.6 MHz for ^19^F detection. The ^19^F chemical shift scale was referenced to trichlorofluoromethane by setting the signal of trifluoroacetic acid in an external reference sample to −76.55 ppm. A single 90° pulse was employed, immediately followed by FID acquisition (zg Bruker pulse program) with a frequency offset of −66.7 ppm and a spectral window of 50.3 ppm. For “closed-tube” cell samples and the corresponding lysates, a set of four spectra with 1280 scans each and an interscan delay of 1 s was recorded on each sample for a total acquisition time of 112 min. The spectra were processed in Topspin with 10 and 5 Hz exponential line broadening for cells and lysates, respectively, phase-corrected, and summed together. A polynomial baseline correction was applied to the sum spectrum to remove a strong baseline distortion arising from polytetrafluoroethylene (PTFE) components inside the probe. For time-resolved in-cell NMR experiments recorded in the bioreactor, a series of ^19^F NMR spectra (512 scans and 1 s delay) was recorded with a time resolution of 11 min/spectrum for an overall duration of up to 66 h. The spectra were processed with 10 Hz exponential line broadening, phased, and baseline-corrected. The time-resolved series were analyzed with the Dynamics Center (Bruker) to measure the peak area as a function of time. The peak areas were corrected to compensate for the gradual loss of Hsp90_N_ in the bioreactor by dividing them by an exponential decay, *e*^–*t*/*T*^, where the time constant *T* was determined from a control experiment (see below). The fraction of Hsp90_N_ bound to the spy ligand was retrieved by dividing the peak area at the plateau after each step of the run (averaged over 2 h) by the peak area measured prior to the addition of the test ligand (averaged over 1 h). Error bars were calculated by error propagation from the standard deviation of the means. ^19^F T_1_ was measured by inversion recovery on cells containing the Hsp90_N_:**2** complex (*T*_1_ = 0.5 ± 0.1 s) and on bioreactor medium containing 20 μM free ligand (*T*_1_ = 0.63 ± 0.08 s). Signal saturation with a 1 s interscan delay was assessed by recording two ^19^F spectra on the above samples with delays of 1 and 10 s, giving integral ratios of 93 and 89%, respectively, indicating that signal saturation does not impact the NMR analysis (Figure S8).

### Curve Fitting

Nonlinear curve fitting was performed with OriginPro 8 (OriginLab) to estimate the rate of intracellular Hsp90_N_ loss in the bioreactor and to retrieve the *K*_d_ of the test ligands relative to that of the spy ligand. The decrease of Hsp90_N_ as a function of time was quantified from the signal of the complex with the spy ligand and was fitted with an exponential decay, resulting in a time constant *T* = 310 ± 30 h (corresponding to a half-life of 215 ± 20 h). The *K*_d_ ratios were obtained by curve fitting with the previously described equation^22,60^
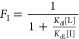
1where *F*_I_ is the fraction of Hsp90_N_ bound to the spy ligand I, [I] and [L] are the concentrations of free spy and the test ligand, respectively, present in the cells, *K*_dI_ and *K*_dL_ are the dissociation constants of I and L, respectively, and *K*_dL_/*K*_dI_ is the *K*_d_ of the test ligand relative to that of the spy ligand. In the NMR bioreactor, intracellular [I] and [L] cannot be measured by NMR due to the low filling factor of the cells in the flow tube and likely due to additional exchange broadening. Because the external solution is continuously replaced, both ligands are in excess and can saturate all intracellular binding sites, so the free ligand concentration inside and outside of the cells is assumed to be equal at equilibrium. Therefore, [I] and [L] are considered equal to the concentration of each ligand in the external solution.

## Supporting Information Available

Target intracellular localization, ^1^H–^15^N in-cell NMR data, ^19^F time-resolved NMR spectra, in-cell NMR leakage control, and ^19^F T_1_ and signal saturation measurements (PDF)
Additional chemistry information related to synthesis and characterization methods and molecular formula strings (CSV)

## Abbreviations USED

CETSA: cellular thermal shift assay
DIAD: diisopropyl azodicarboxylate
DMEM: Dulbecco-modified Eagle medium
FBS: fetal bovine serum
FID: free induction decay
FP: fluorescence polarization
GI_50_: growth inhibitory dose of 50%
HATU: hexafluorophosphate azabenzotriazole tetramethyl uranium
HBTU: hexafluorophosphate benzotriazole tetramethyl uronium
HEPES: 2-[4-(2-hydroxyethyl)piperazin-1-yl]ethanesulfonic acid
Hsp90α: 90 kDa heat shock protein alpha
Hsp90_N_: N-terminal domain of Hsp90α
*K*_d_: dissociation constant
MEM: 2-methoxyethoxymethyl ether
PEEK: polyether ether ketone
PEI: polyethylenimine
PPTS: pyridinium *p*-toluenesulfonate
PTFE: polytetrafluoroethylene
SOFAST-HMQC: band-selective optimized flip angle short transient heteronuclear multiple quantum correlation
TBAF: *tert* butyl ammonium fluoride
TFA: trifluoroacetic acid
U–^15^N: uniform-^15^N
