# Supplementary material for: Ligand-Based Competition Binding by Real-Time 19F NMR in Human Cells
Source: J Med Chem. 2024 Jan 12;67(2):1115–26. doi: 10.1021/acs.jmedchem.3c01600 (PMC10823471; doi:10.1021/acs.jmedchem.3c01600)
Supplement: Supplementary file 1 — jm3c01600_si_001.pdf [file jm3c01600_si_001.pdf]

**Supplementary Information**

# Ligand-based competition binding by real-time $^{19}$F NMR in human cells

Enrico Luchinat[1,2,*], Letizia Barbieri[2], Ben Davis[3], Paul A. Brough[3], Matteo Pennestri[4], Lucia Banci[2,5,*]

[1] Dipartimento di Scienze e Tecnologie Agro-Alimentari, Alma Mater Studiorum – Università di Bologna, Piazza Goidanich 60, 47521 Cesena, Italy;
[2] Consorzio Interuniversitario Risonanze Magnetiche di Metallo Proteine – CIRMMP, Via Luigi Sacconi 6, 50019 Sesto Fiorentino, Italy;
[3] Vernalis Research, Granta Park, Great Abington, Cambridge, CB21 6GB, United Kingdom;
[4] Pharmaceutical Business Unit, Bruker UK Limited, Banner Lane, Coventry, CV4 9GH, United Kingdom;
[5] Centro di Risonanze Magnetiche – CERM, Università degli Studi di Firenze, Via Luigi Sacconi 6, and Dipartimento di Chimica, Università degli Studi di Firenze, Via della Lastruccia 3, 50019 Sesto Fiorentino, Italy;
* Email: enrico.luchinat@unibo.it; banci@cerm.unifi.it

**TABLE OF CONTENTS** **page**

- **Supplementary Figures S1-S8** S2
- **Supplementary Table S1** S11
- **Chemistry**
  - General Procedures S12
    - **Table S2** S13
    - **Table S3** S14
  - Compounds **1, 3** and **5** S15
    - **Scheme S1** S15
    - **Figure S9** S16
    - **Figure S10** S17
    - **Figure S11** S18
  - Compounds **2** and **6** S19
    - **Scheme S2** S19
    - **Figure S12** S20
    - **Figure S13** S21
  - Compounds **4** and **7** S22
    - **Scheme S3** S22
    - **Figure S14** S23
    - **Figure S15** S24
    - **Table S4** S25
- **References** S26

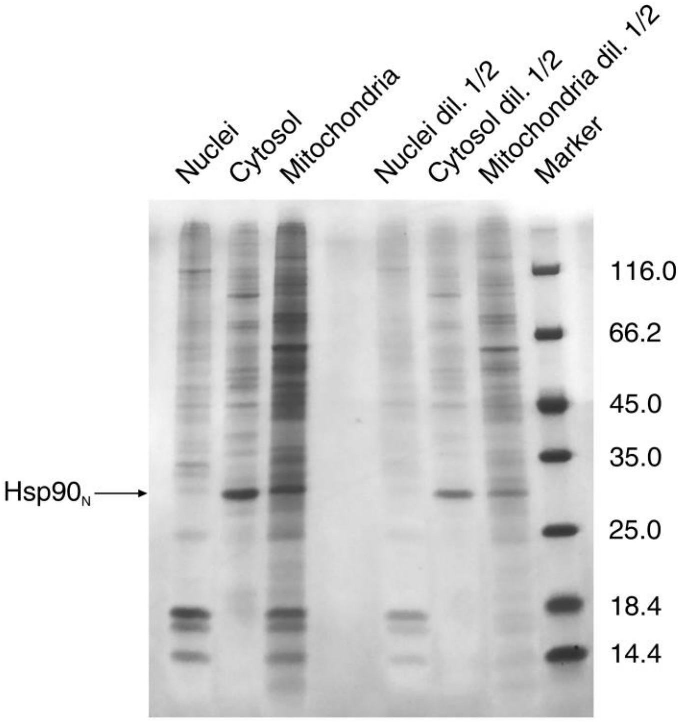


**Figure S1. Intracellular localization of Hsp90$_N$.** SDS-PAGE analysis of the nuclear, cytosolic and mitochondrial fractions of HEK293T cells overexpressing Hsp90$_N$. The nuclear and cytosolic fractions are diluted 1:10 and 1:40 with respect to the mitochondrial fraction, respectively.

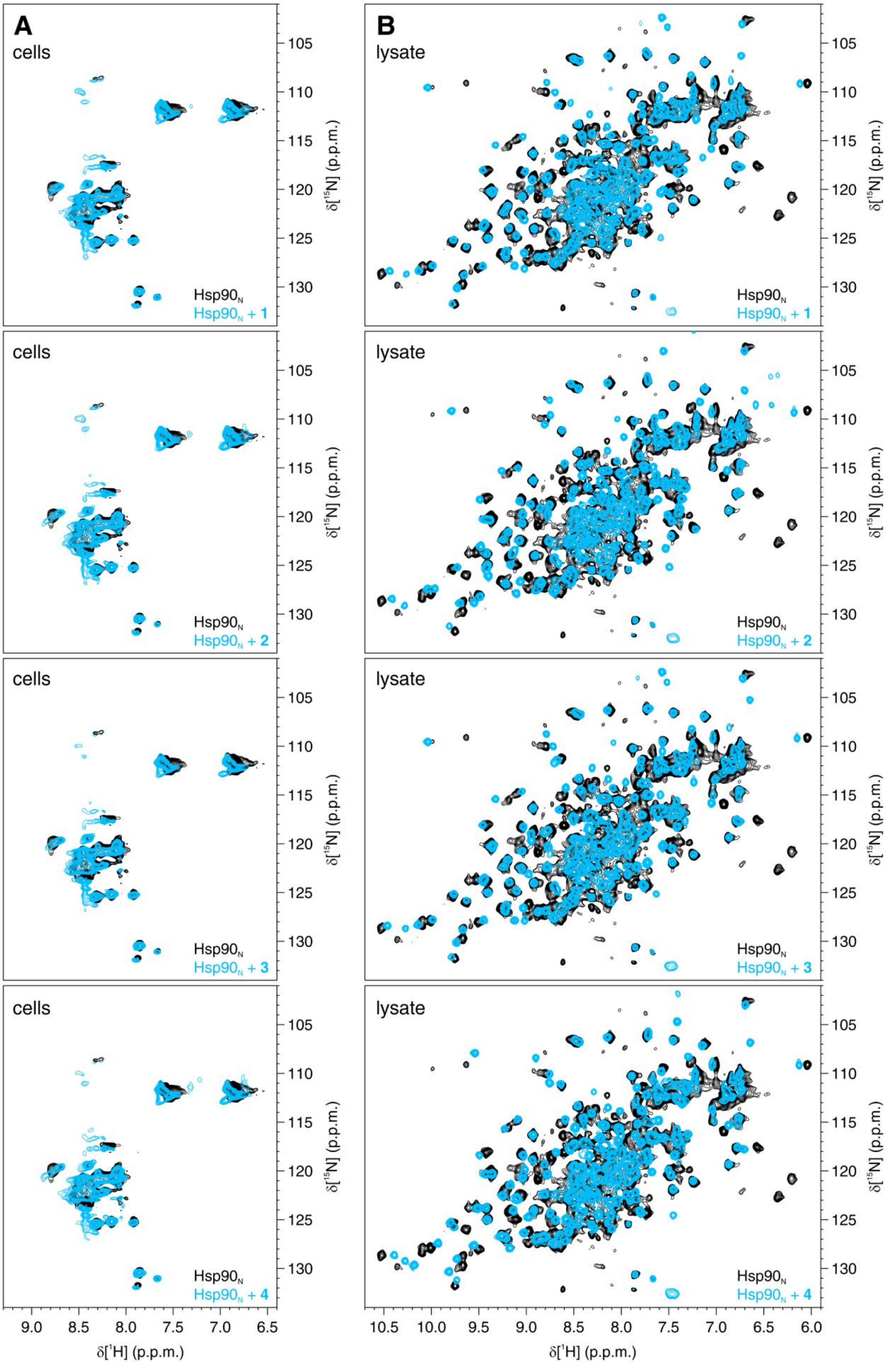
A
cells
Hsp90N
Hsp90N + 1
cells
Hsp90N
Hsp90N + 2
cells
Hsp90N
Hsp90N + 3
cells
Hsp90N
Hsp90N + 4
B
lysate
Hsp90N
Hsp90N + 1
lysate
Hsp90N
Hsp90N + 2
lysate
Hsp90N
Hsp90N + 3
lysate
Hsp90N
Hsp90N + 4
δ[15N] (p.p.m.)
δ[1H] (p.p.m.)

**Figure S2. Binding of compounds 1-4 to intracellular Hsp90$_N$.** Background-subtracted $^1H$-$^{15}N$ SOFAST-HMQC spectra of human cells (A) and corresponding lysates (B) expressing [U-$^{15}N$]-Hsp90$_N$ in the absence (black) and in the presence (blue) of a fluorinated compound ( **1**-**4** from top to bottom). Ligand binding is revealed by the shifted crosspeaks in the $^1H$-$^{15}N$ NMR spectra of the cell lysates.

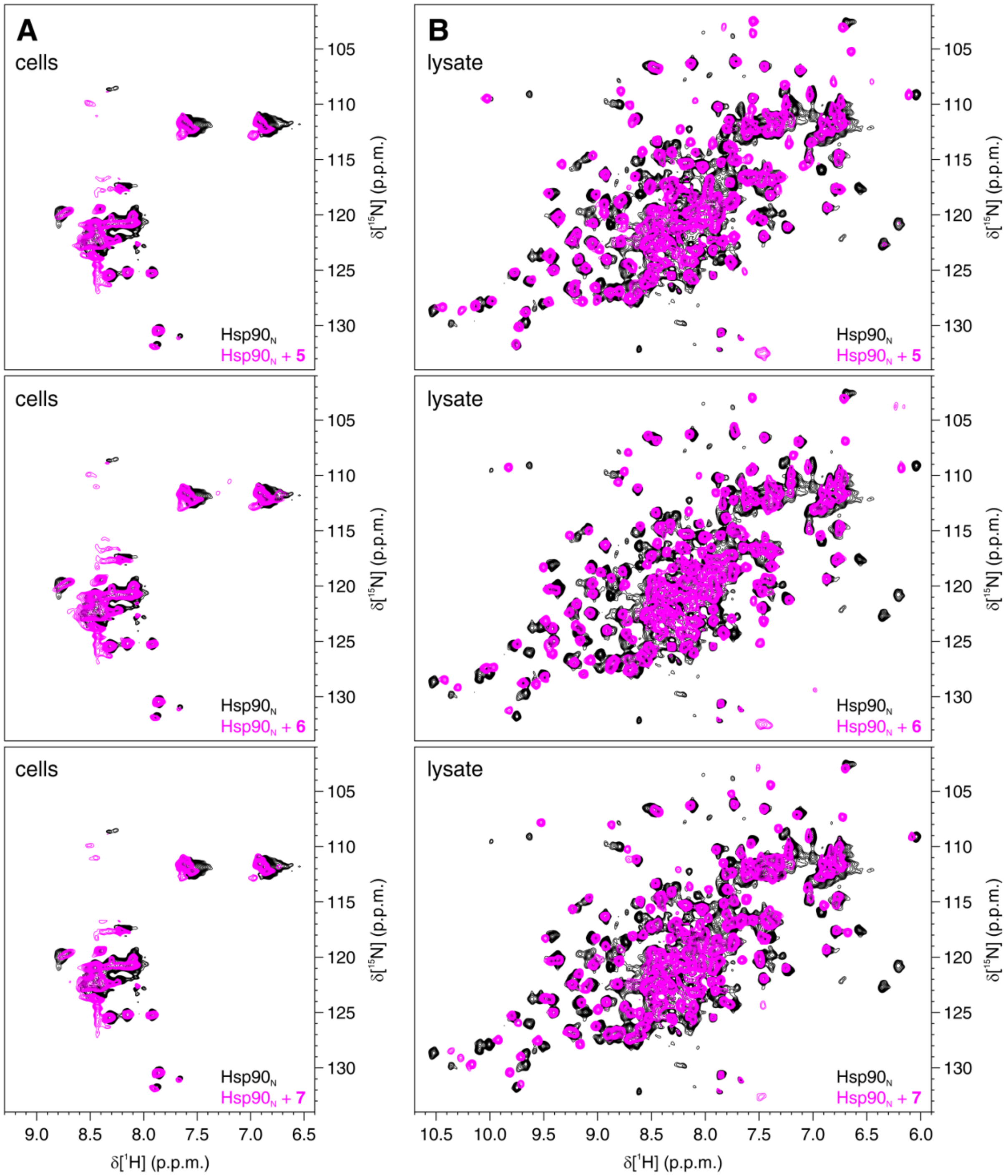


**Figure S3. Binding of compounds 5-7 to intracellular Hsp90$_N$.** Background-subtracted $^1$H-$^{15}$N SOFAST-HMQC spectra of human cells (A) and corresponding lysates (B) expressing [U-$^{15}$N]-Hsp90$_N$ in the absence (black) and in the presence (magenta) of a test compound (**5**-**7** from top to bottom). Ligand binding is revealed by the shifted crosspeaks in the $^1$H-$^{15}$N NMR spectra of the cell lysates.

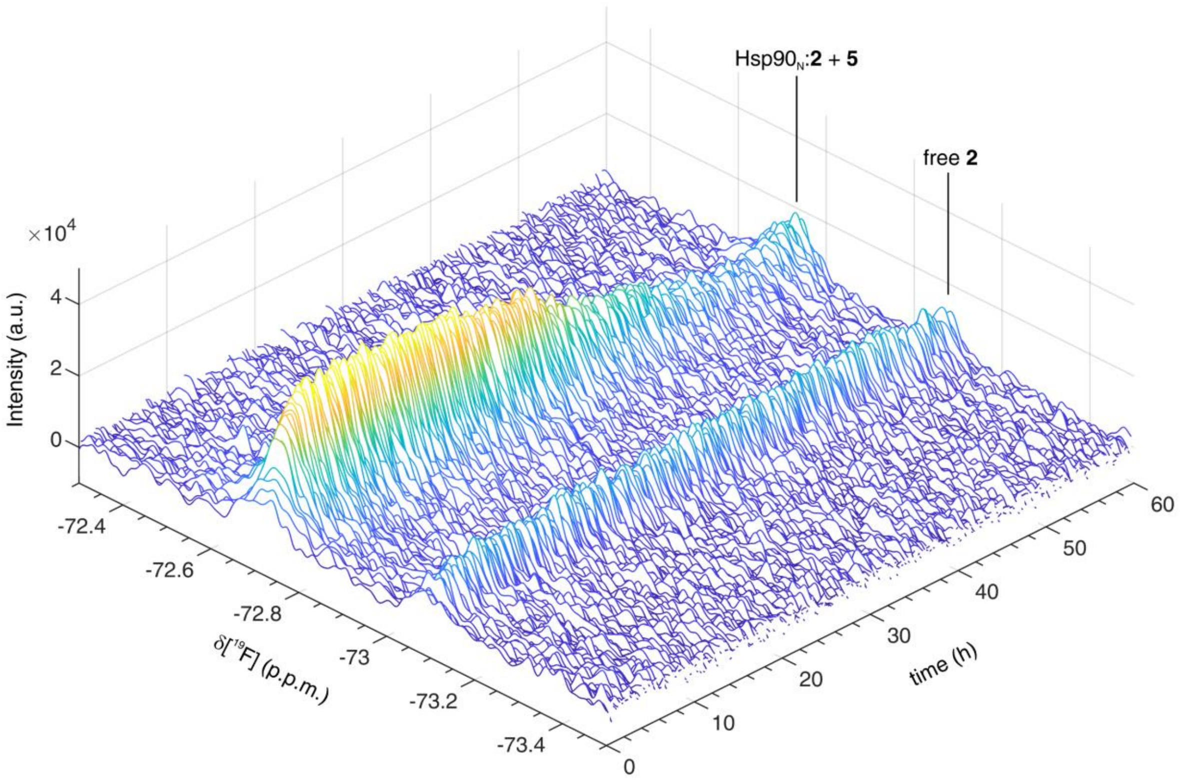


**Figure S4. Competition binding of compound 5.** Waterfall plot of time-resolved 1D $^{19}$F NMR spectra recorded on cells expressing Hsp90$_N$, perfused in the bioreactor with a constant concentration of compound **2** at increasing concentrations of compound **5** over the course of ~60 hours. Spectral intensity (a.u.) is color-coded from blue (lowest) to yellow (highest). Hsp90$_N$:**2** + **5**: signal arising from the Hsp90$_N$:**2** complex as it is displaced by compound **5**; free **2**: signal arising from free compound **2** in the extracellular medium.

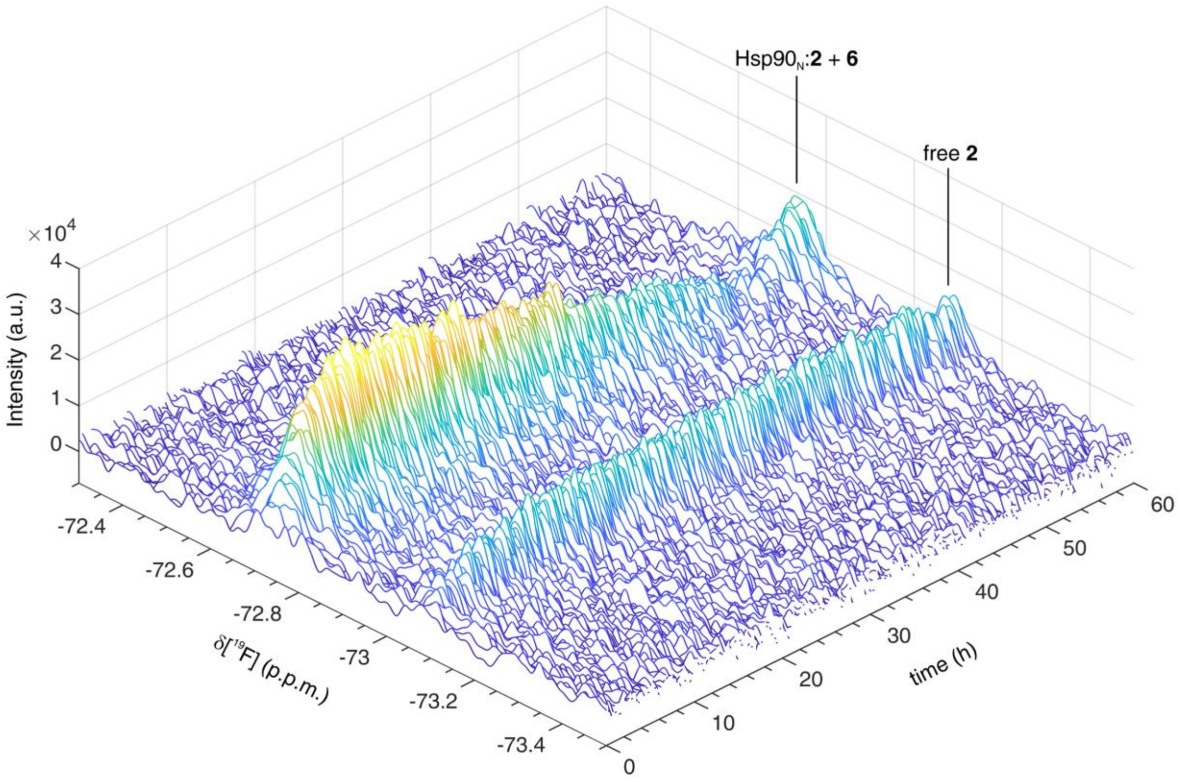


**Figure S5. Competition binding of compound 6.** Waterfall plot of time-resolved 1D $^{19}$F NMR spectra recorded on cells expressing Hsp90$_N$, perfused in the bioreactor with a constant concentration of compound **2** at increasing concentrations of compound **6** over the course of ~60 hours. Spectral intensity (a.u.) is color-coded from blue (lowest) to yellow (highest). Hsp90$_N$:**2** + **6**: signal arising from the Hsp90$_N$:**2** complex as it is displaced by compound **6**; free **2**: signal arising from free compound **2** in the extracellular medium.

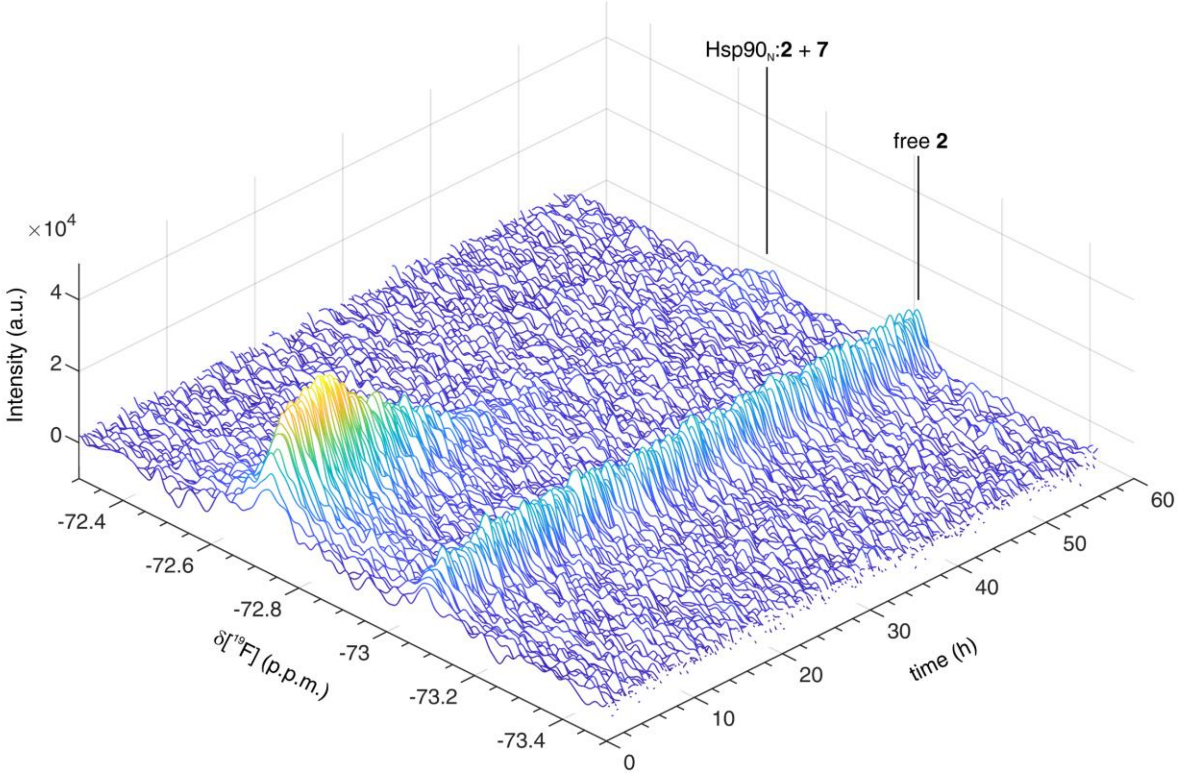


**Figure S6. Competition binding of compound 7.** Waterfall plot of time-resolved 1D $^{19}$F NMR spectra recorded on cells expressing Hsp90$_N$, perfused in the bioreactor with a constant concentration of compound **2** at increasing concentrations of compound **7** over the course of ~56 hours. Spectral intensity (a.u.) is color-coded from blue (lowest) to yellow (highest). Hsp90$_N$:**2** + **7**: signal arising from the Hsp90$_N$:**2** complex as it is displaced by compound **7**; free **2**: signal arising from free compound **2** in the extracellular medium.

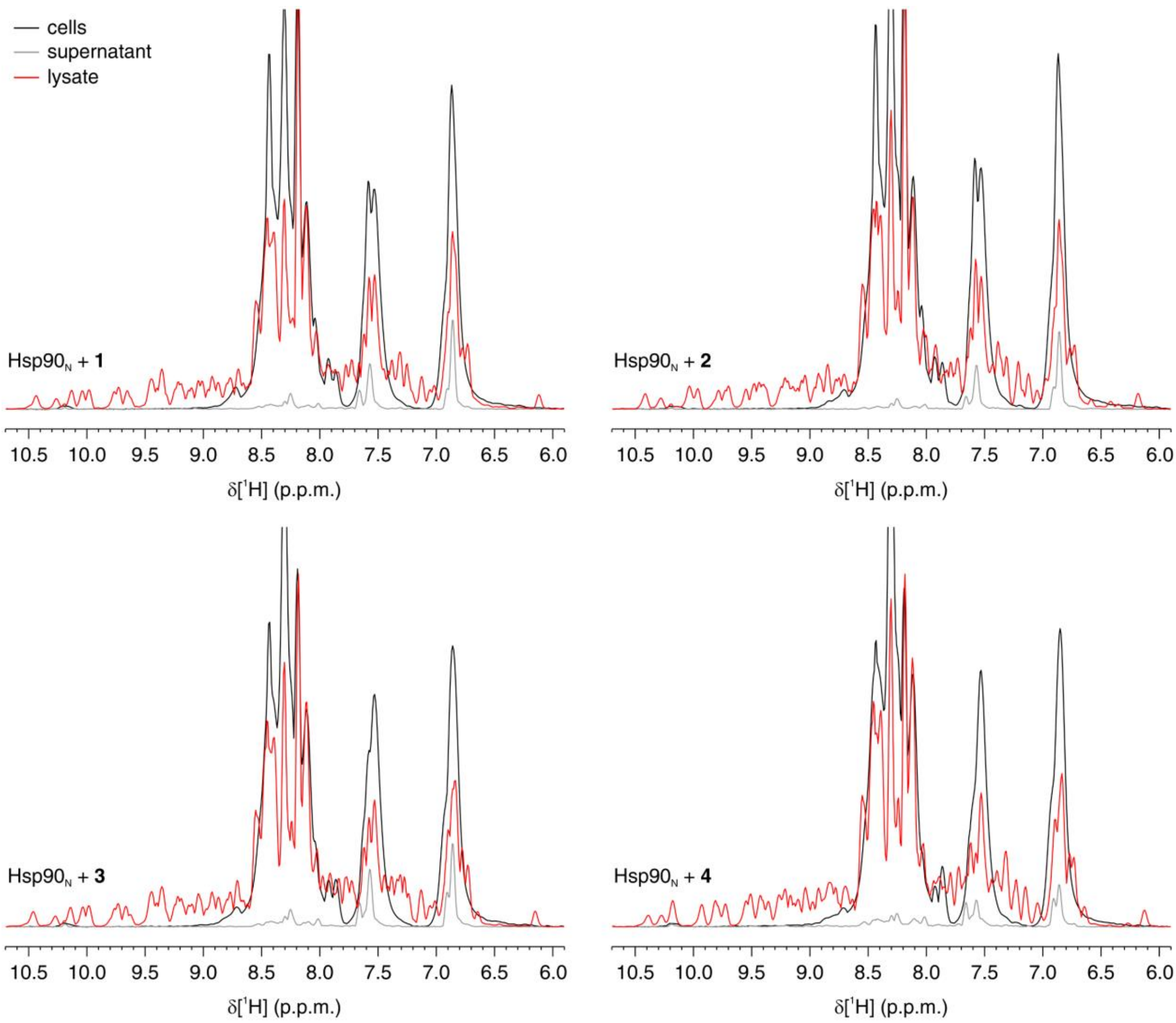


**Figure S7. In-cell, leakage control and lysate NMR spectra.** Projections along the $^{1}H$ axis of $^{1}H$-$^{15}N$ SOFAST-HMQC spectra of cells (black), supernatants (grey) and cell lysates (red) from samples of cells expressing $Hsp90_N$ treated with compounds **1**-**4**.

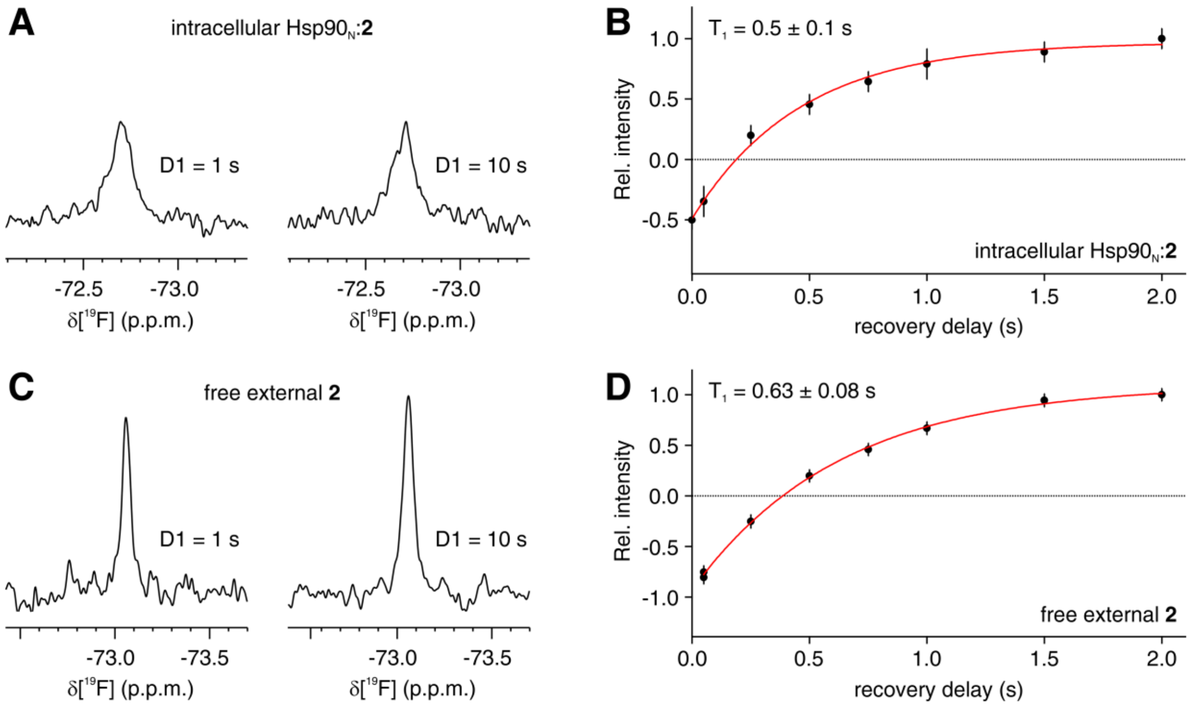


**Figure S8. Saturation of the $^{19}$F signals.** (A) Signal arising from the intracellular Hsp90$_N$:**2** complex recorded with an interscan delay (D1) of 1 s (left) and 10 s (right); (B) $T_1$ relaxation analysis of Hsp90$_N$:**2** by inversion recovery; (C) Signal arising from free compound **2** in DMEM medium recorded with an interscan delay (D1) of 1 s (left) and 10 s (right); (D) $T_1$ relaxation analysis of compound **2** by inversion recovery.

**Table S1. Durations and ligand concentrations of each step of the bioreactor runs reported in Figure 4A-C.** For each channel, ligand concentrations in the reservoir and flow rates are also reported.

| **Bioreactor run 1 (Figure 4A)** – Ch. 1: **2** 10 µM; Ch. 2: **2** 10 µM + **5** 20 µM | | | | | |
|---|---|---|---|---|---|
| Step | Duration (h) | **2** (µM) | **5** (µM) | Ch. 1 flow rate (µL/min) | Ch. 2 flow rate (µL/min) |
| 1 | 6 | 10 | 0 | 100 | 0 |
| 2 | 12 | 10 | 1 | 95 | 5 |
| 3 | 12 | 10 | 2 | 90 | 10 |
| 4 | 12 | 10 | 5 | 75 | 25 |
| 5 | 6 | 10 | 10 | 50 | 50 |
| 6 | 6 | 10 | 20 | 0 | 100 |
| 7 | 12 | 10 | 0 | 100 | 0 |
| **Bioreactor run 2 (Figure 4B)** – Ch. 1: **2** 10 µM; Ch. 2: **2** 10 µM + **6** 20 µM | | | | | |
| Step | Duration (h) | **2** (µM) | **6** (µM) | Ch. 1 flow rate (µL/min) | Ch. 2 flow rate (µL/min) |
| 1 | 6 | 10 | 0 | 100 | 0 |
| 2 | 12 | 10 | 1 | 95 | 5 |
| 3 | 12 | 10 | 2 | 90 | 10 |
| 4 | 12 | 10 | 5 | 75 | 25 |
| 5 | 6 | 10 | 10 | 50 | 50 |
| 6 | 6 | 10 | 20 | 0 | 100 |
| 7 | 12 | 10 | 0 | 100 | 0 |
| **Bioreactor run 3 (Figure 4C)** – Ch. 1: **2** 10 µM; Ch. 2: **2** 10 µM + **7** 20 µM | | | | | |
| Step | Duration (h) | **2** (µM) | **7** (µM) | Ch. 1 flow rate (µL/min) | Ch. 2 flow rate (µL/min) |
| 1 | 6 | 10 | 0 | 100 | 0 |
| 2 | 12 | 10 | 1 | 95 | 5 |
| 3 | 12 | 10 | 2 | 90 | 10 |
| 4 | 12 | 10 | 5 | 75 | 25 |
| 5 | 15 | 10 | 0 | 100 | 0 |

**Chemistry**

General procedures

$^1H$ (400 MHz) and $^{13}C$ (100.6 MHz) Nuclear magnetic resonance (NMR) analyses were performed using a Bruker DPX-400 MHz NMR spectrometer. $^1H$ NMR Spectra were also recorded at 250 MHz on a Bruker AC250 and at 500 MHz on a Bruker 500 MHz Ultrashield spectrometer. The spectral reference was the known chemical shift of the sample solvent. 1H NMR data is reported indicating the chemical shift (d) as parts per million (ppm), the multiplicity, (s, singlet; d, doublet; t, triplet; q, quartet; sept, septet; m, multiplet; br, broad; dd, doublet of doublets etc.) the integration (e.g. 1H), the coupling constant (*J*) in Hertz (Hz) (app implies apparent coupling on broadened signals). 13C NMR data is reported indicating the chemical shift (d) as parts per million (ppm), and i n some cases annotated with the carbon multiplicity: (CH3) for primary carbon, (CH2) for secondary carbon, (CH) for tertiary carbon and (C) for quaternary carbon. Deuterated solvents were obtained from the Sigma-Aldrich Chemical Company or Fluorochem. LCMS analyses were performed on an HP1100 instrument (*method A*), with a Luna 3 DM, C18(2), 30 mm × 4.6 mm i.d. column from Phenomenex at a temperature of 22 °C, with a flow rate of 2 mL min-1 using the following solvent systems (solvents purchased from Romil UK, Waterbeach, UK; Solvent A: HPLC grade water + 10 mM ammonium acetate + 0.08% v/v formic acid. Solvent B: 95% v/v HPLC grade acetonitrile + 5% v/v Solvent A + 0.08% v/v formic acid. Gradient: 95:5 Solvent A : Solvent B, 0.00 to 0.25 mins; 95 : 5 to 5 : 95 Solvent A : Solvent B, 0.25 to 2.50 mins; 5 : 95 Solvent A : Solvent B, 2.50 to 3.75 mins. UV detection was at 230 nm, 254 nm and 270 nm. The mass spectrometer was an HP1100MSD, Series A instrument, operating in positive or negative ion electrospray ionization mode. Molecular weight scan range is 120 to 1000. Samples were supplied as a 1 mM solution in DMSO, with 5 DL partial loop fill injection. LC purities were assigned one of three values; 85-90%; 90-95% or >95%. Chemical samples were also analyzed by a separate LCMS system (*method B*) using a Micromass LCT / Water's Alliance 2795 HPLC system with a Discovery 5 Dm, C18, 50 mm × 4.6 mm i.d. column from Supelco at a temperature of 22°C using the following solvent systems: Solvent A: MeOH; Solvent B: 0.1% Formic acid in water at a flow rate of 1 mL / min. Gradient starting with 10% A : 90% B from 0 - 0.5 mins then 10% A : 90% B to 90% A : 10% B from 0.5 mins to 6.5 mins and continuing at 90% A : 10% B up to 10 mins. From 10-10.5 mins the gradient reverted back to 10% A : 90% where the concentrations remained until 15 mins. UV detection was at 254 nm and ionization was positive or negative ion electrospray. Molecular weight scan range is 50-1000. Samples were supplied as 1mg/mL in DMSO or MeOH with 3mL injected on a partial loop fill.

**HPLC analysis (method A).** HPLC was performed on a Perkin Elmer series 200 quaternary pump and 235C DAD instrument, with a Gemini 5 DM, C18 110A 50 mm × 4.6 mm i.d. column from Phenomenex (part number 00B-4435-E0) at a temperature of 22 °C, at a flow rate of 2 mL min-1 using the following solvent systems (Solvents purchased from Romil UK, Waterbeach, UK) Solvent A: HPLC grade Water + 10 mM ammonium acetate adjusted to pH 7.5 with ammonium hydroxide. Solvent B: 100% acetonitrile. Gradient: 80:20 Solvent A: Solvent B, 0.00 to 2.0 mins; 80 : 20 to 5 : 95 Solvent A : Solvent B, 2.0 mins to 8.0 mins; 5 : 95 Solvent A : Solvent B, 8.0 to 11.5 mins. UV detection was at 225 nm, injection volume: 5 mL.

**HPLC analysis (method B).** HPLC was performed on an Agilent 1200SL series instruments connected to an Agilent MSD 6140 single quadrupole with a multimode source. The column was a Luna 2.5mm C18, 50x2 mm, HST from Phenomenex. at a temperature of 55 °C. The solvents used and gradient / flow rates are shown below (Table S2):- Mobile phase: Solvent **A**: Water /10 mM ammonium formate / 0.04% (v/v) formic acid pH=3.5. Solvent **B:** Acetonitrile / 5.3 % (v/v) A / 0.04% (v/v) formic. Injection volume: 2 mL.

**Table S2**: Solvent gradient used for analytical HPLC method B.

| TIME (min) | Solvent A (%) | Solvent B (%) | FLOW |
|---|---|---|---|
| 0.00 | 95 | 5 | 1.1 |
| 0.12 | 95 | 5 | 1.1 |
| 1.30 | 5 | 95 | 1.1 |
| 1.35 | 5 | 95 | 1.7 |
| 1.85 | 5 | 95 | 1.7 |
| 1.90 | 5 | 95 | 1.1 |
| 1.95 | 95 | 5 | 1.1 |

Signal Detection was by UV at 254 nm.

Flash chromatography was performed using pre-packed silica gel cartridges (Strata Si-1, 61 Å from Phenomenex, Cheshire UK, or IST Flash II, 54 Å from Argonaut, Hengoed UK). High Resolution Mass Spectrum analyses and combustion analyses were performed by Medac Ltd, Egham, Surry, UK. High Resolution Mass Spectrum analyses were additionally performed as follows:

| | |
|---|---|
| Instrument: | Agilent 1290 Infinity II UHPLC series connected to a TOF 6230 with an ES ionization source (positive or negative mode ionization). |
| Column: | Kinetex C18, 2.6 microns, 50x2 mm, (Phenomenex). |
| Temperature: | 55 °C. |
| Mobile phase: | A: Water /10 mM ammonium formate / 0.08% (v/v) formic acid pH=3.3. |
| | B: Acetonitrile / 5.3 % (v/v) A / 0.08% (v/v) formic. |
| Flow: | 1.3 mL/min. |
| Injection volume: | 1 mL. |
| Gradient: | From 5 to 95 % in 1.18min, run time 1.95 min. |

Thin layer chromatography was conducted with 5 × 10 cm plates coated with Merck Type 60 F254 silica gel to a thickness of 0.25mm. All reagents obtained from commercial sources were used without further purification. Anhydrous solvents were obtained from the Sigma-Aldrich Chemical Company Ltd., and used without further drying.

**PREPARATIVE HPLC.** Preparative HPLC purifications were performed on a Waters FractionLynx MS Autopurification system with a Gemini® 5 µM C18(2), 100 mm × 20 mm i.d. column from Phenomenex, running at a flow rate of 20 mL min-1 with UV diode array detection (210 – 400 nm) and mass-directed collection. Representative Gradients used are shown in Table S3.

**At pH 4**:
Solvent **A**: HPLC grade Water + 10mM ammonium acetate + 0.08% v/v formic acid.
Solvent **B**: 95% v/v HPLC grade acetonitrile + 5% v/v Solvent A + 0.08% v/v formic acid.

**At pH 9**:
Solvent A: HPLC grade Water + 10 mM ammonium acetate + 0.08% v/v ammonia solution.
Solvent **B**: 95% v/v HPLC grade acetonitrile + 5% v/v Solvent A + 0.08% v/v ammonia solution.

The mass spectrometer was a Waters Micromass ZQ2000 spectrometer operating in positive or negative ion electrospray ionization modes, with a molecular weight scan range of 150 to 1000.

**Table S3.** Preparative HPLC gradients, showing 4 representative gradients (W-Z) used for purifying certain compounds described below.

| Time /min | % solvent **B** for gradient W-Z. | | | |
|---|---|---|---|---|
| | **W** | **X** | **Y** | **Z** |
| 0.0 | 5 | 5 | 5 | 5 |
| 0.5 | 20 | 25 | 30 | 35 |
| 7.0 | 40 | 45 | 50 | 55 |
| 7.5 | 95 | 95 | 95 | 95 |
| 9.5 | 95 | 95 | 95 | 95 |
| 10 | 5 | 5 | 5 | 5 |

Compounds **1**, **3** and **5**

The synthesis and analytical methods for pertaining to compounds **3** and **5** is described in detail elsewhere.[1] Compound **1** is synthesized by a directly analogous method to compound **3.** Compounds **1**,**3** and **5** are prepared by the synthetic procedures outlined in Scheme S1.

**Scheme S1.** Synthetic procedure utilized for preparation of 2-aminothieno[2,3-*d*]pyrimidine inhibitors.[a] Adapted with permission from Brough, P. A. et al . J Med Chem 2009, 52 (15), 4794–4809. Copyright 2009, ACS.

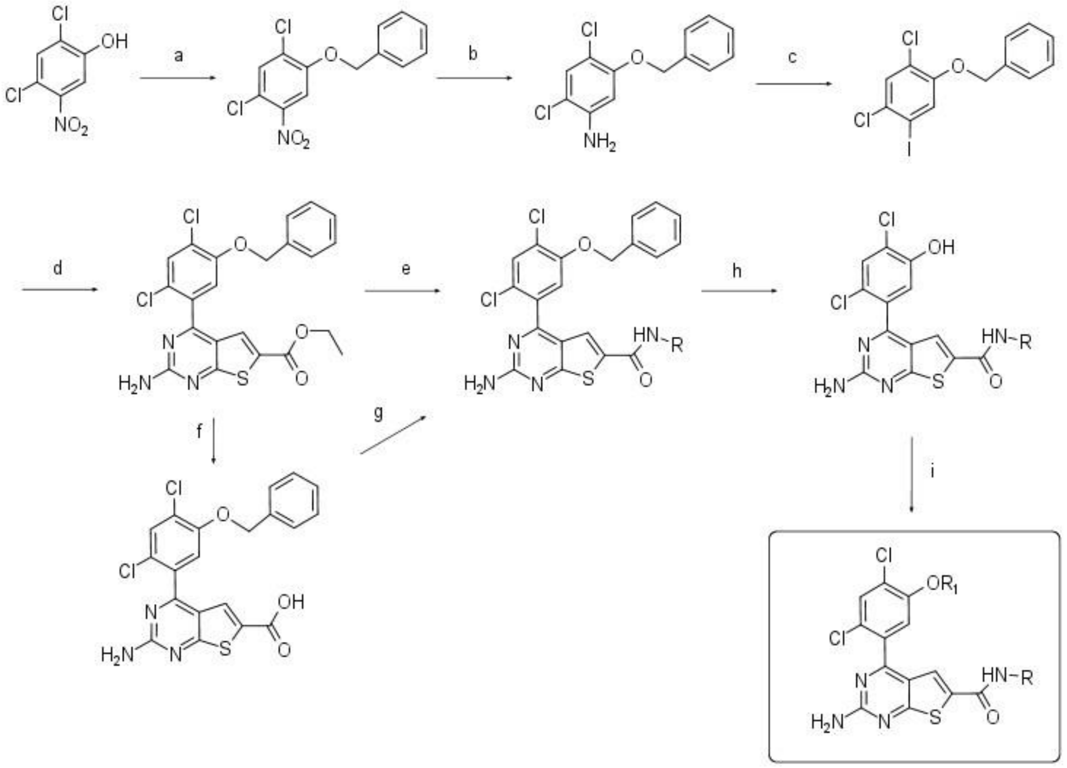


[a]Reagents and conditions: (a) BnBr, $K_2CO_3$, MeCN, Δ, 18 h, 96%; (b) Iron powder, AcOH, $H_2O$, 85 °C, 1.5 h, 96%; (c) (*i*) $NaNO_2$, HCl, $H_2O$, AcOH, 0 to 5 °C, 30 min, (*ii*) $I_2$, KI, $H_2O$, rt,1.5 h, 90%; (d) (*i*) KOAc, bis(pinacolato)diboron, $Pd(OAc)_2$, DMF, 90 °C, 18 h, (*ii*) 2-Amino-4-chloro-thieno[2,3-d]pyrimidine-6-carboxylic acid ethyl ester, $K_3PO_4$, 1,4-dioxane, $Pd(PPh_3)_2Cl_2$, 100 °C, 3 h, 45%; (e) for R=Et, $EtNH_2$, MeOH, Δ, 18 h, 84%; (f) NaOH, $H_2O$, EtOH, D, 50 min., quant.; (g) HATU, $RNH_2$, DMF, diisopropylamine; 50 °C, 1 h; (h) $BCl_3$, $CH_2Cl_2$, -78 °C to rt, 3 h, 87%; (i) $R_1$Br/Cl, $Cs_2CO_3$, DMF, 100-140 °C, 1-2 h; or $R_1$OH, DIAD, $PPh_3$, THF, rt, 4 h.

**2-Amino-4-[2,4-dichloro-5-(2-diethylamino-ethoxy)-phenyl]-thieno[2,3-d]pyrimidine-6-carboxylic acid (2,2,2-trifluoro-ethyl) amide (3)**

Compound **3** , off-white powder: LCMS $t_R$ = 2.02 min; *m/z* = 538, 536 $[M+H]^+$; $^1$H NMR (400 MHz, DMSO-$d_6$) δ 0.95 (t, 6H, *J* = 7.1 Hz), 2.54 (q, 4H, *J* = 7.1 Hz), 2.80 (t, 2H, *J* = 5.7 Hz), 4.00-4.09 (m, 2H), 4.12 (t, 2H, *J* = 5.7 Hz), 7.36 (brs, 2H), 7.40 (s, 1H), 7.76 (s, 1H), 7.83 (s, 1H), 9.17 (brt, 1H, *J* = 6.3 Hz); $^{19}$F NMR (376 MHz, DMSO-$d_6$) δ -70.50; $^{13}$C NMR (100.6 MHz, DMSO-$d_6$) d 11.8 ($CH_3$), 39.9 ($CH_2$, obscured by DMSO), 47.1 ($CH_2$), 50.8 ($CH_2$), 68.2 ($CH_2$), 115.3 (CH), 121.1 (C), 122.3 (C), 123.1 (C), 123.2 (CH), 124.6 (C, q, $J_{C-F}$ = 280 Hz), 130.4 (CH), 130.4 (C), 135.5 (C), 153.0 (C), 161.3 (C), 161.8 (C), 162.0 (C), 171.4 (C); HRMS, calcd for $C_{21}H_{22}Cl_2F_3N_5O_2S$ $[M+H]^+$ 536.0902 found 536.0912; HPLC (*method A*) 97.3% ($t_R$ = 4.64 min).

Page 1 of 1

Software Version : 6.3.0.0445
Sample Name : 83551-1
Instrument Name : DIODE
Rack/Vial : 0/24
Sample Amount : 1.000000
Cycle : 5

Date : 21/02/2008 09:57:14
Data Acquisition Time : 20/02/2008 16:04:03
Channel : A
Operator : tim
Dilution Factor : 1.000000

Result File : \\server4\tcdata\LC Data\DIODE_20_02_2008_A_024.rst
Sequence File : \\server4\TCData\LC Sequences\DIODE 2008 FEB 18.seq

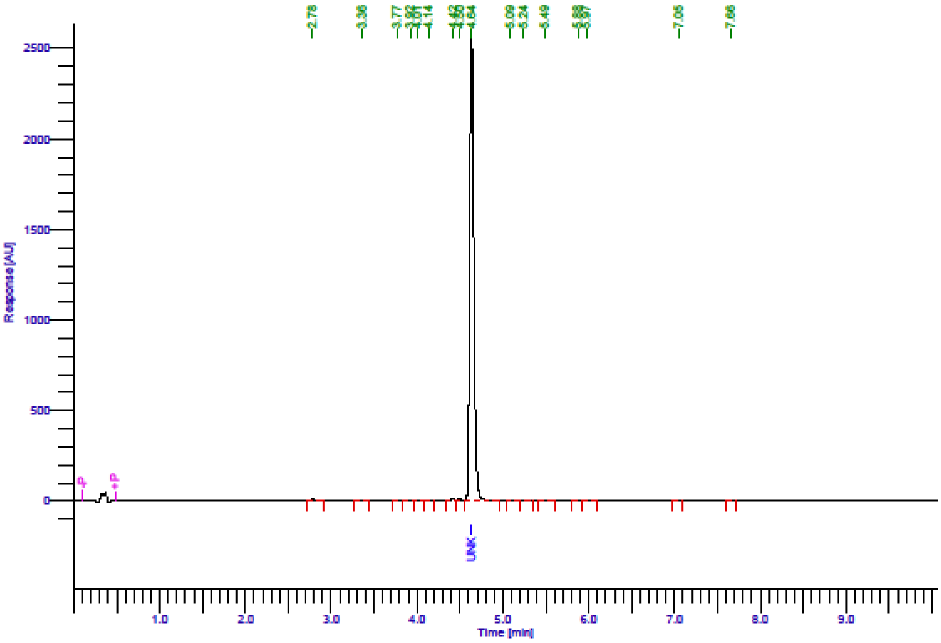


20% to 95% gradient GEMINI 2.0 ml/min Acn/H20

Wavelength 225 BW15nm, 5ul inj

| Peak # | Time [min] | Area [uV*sec] | Height [uV] | Area [%] | Norm. Area [%] | BL | Area/Height [sec] |
|---|---|---|---|---|---|---|---|
| 1 | 2.784 | 29101.69 | 9630.81 | 0.33 | 0.33 | BB | 3.0217 |
| 2 | 3.361 | 10873.49 | 4710.11 | 0.13 | 0.13 | BB | 2.3085 |
| 3 | 3.765 | 4742.37 | 1496.28 | 0.05 | 0.05 | BV | 3.1694 |
| 4 | 3.923 | 13886.30 | 3783.81 | 0.16 | 0.16 | VV | 3.6699 |
| 5 | 4.006 | 23573.08 | 6841.66 | 0.27 | 0.27 | VB | 3.4455 |
| 6 | 4.137 | 8727.38 | 2723.91 | 0.10 | 0.10 | BB | 3.2040 |
| 7 | 4.420 | 46367.02 | 15066.74 | 0.53 | 0.53 | BV | 3.0774 |
| 8 | 4.499 | 36010.17 | 12017.07 | 0.41 | 0.41 | VV | 2.9966 |
| 9 | 4.635 | 8463637.94 | 2.55e+06 | 97.34 | 97.34 | VB | 3.3135 |
| 10 | 5.088 | 13943.16 | 4332.41 | 0.16 | 0.16 | BV | 3.2183 |
| 11 | 5.243 | 9612.22 | 2823.60 | 0.11 | 0.11 | VB | 3.4042 |
| 12 | 5.492 | 7191.44 | 1262.90 | 0.08 | 0.08 | BB | 5.6944 |
| 13 | 5.875 | 6235.85 | 1654.72 | 0.07 | 0.07 | BV | 3.7685 |
| 14 | 5.968 | 12727.52 | 2299.36 | 0.15 | 0.15 | VB | 5.5353 |
| 15 | 7.049 | 3118.37 | 1027.93 | 0.04 | 0.04 | BB | 3.0337 |
| 16 | 7.660 | 5239.87 | 1104.38 | 0.06 | 0.06 | BB | 4.7446 |
| | | 8694987.87 | 2.63e+06 | 100.00 | 100.00 | | |

Warning – Signal level out-of-range in peak

Missing Component Report
Component Expected Retention (Calibration File)

All components were found

**Figure S9.** HPLC trace for compound **3**.

**2-Amino-4-[2,4-dichloro-5-(2-diethylamino-ethoxy)-phenyl]-thieno[2,3-d]pyrimidine-6-carboxylic acid ethylamide (5)**

compound **5**, off-white powder: TLC $R_f$ = 0.10 (DCM/MeOH 9:1); LCMS $t_R$ = 1.86 min; *m/z* = 484, 482 $[M+H]^+$; $^1$H NMR (400 MHz, DMSO-$d_6$) δ 0.95 (t, 6H, *J* = 7.1 Hz), 1.07 (t, 3H, *J* = 7.2 Hz), 2.54 (q, 4H, *J* = 7.1 Hz), 2.80 (brt, 2H, *J* = 5.5 Hz), 3.21 (m, 2H), 4.12 (brt, 2H, *J* = 5.5 Hz), 7.27 (brs, 2H), 7.38 (s, 1H), 7.58 (s, 1H), 7.82 (s, 1H), 8.56 (brt, 1H, *J* = 5.5 Hz); $^{13}$C NMR (100.6 MHz, DMSO-$d_6$) δ 12.0 ($CH_3$), 14.7 ($CH_3$), 34.0 ($CH_2$), 47.1 ($CH_2$), 50.9 ($CH_2$), 68.4 ($CH_2$), 115.3 (CH), 121.3 (C), 121.3 (CH), 122.3 (C), 123.0 (C), 130.3 (CH), 132.6 (C), 135.6 (C), 153.0 (C), 160.8 (C), 161.1 (C), 161.5 (C), 171.1 (C); HRMS, calcd for $C_{21}H_{25}Cl_2N_5O_2S$ $[M+H]^+$ 482.1184, found 482.1196; HPLC (*method A*) 99.3% ($t_R$ = 4.68 min).

Page 1 of 1

| | | | |
|---|---|---|---|
| Software Version | : 6.3.0.0445 | Date | : 31/01/2008 11:34:11 |
| Sample Name | : 82160-005 | Data Acquisition Time | : 31/01/2008 10:08:52 |
| Instrument Name | : DIODE | Channel | : A |
| Rack/Vial | : 0/2 | Operator | : tim |
| Sample Amount | : 1.000000 | Dilution Factor | : 1.000000 |
| Cycle | : 2 | | |

Result File : \\server4\tcdata\LC Data\DIODE_31_01_2008_A_002.rst
Sequence File : \\server4\TCData\LC Sequences\DIODE 2008 FEB 01.seq

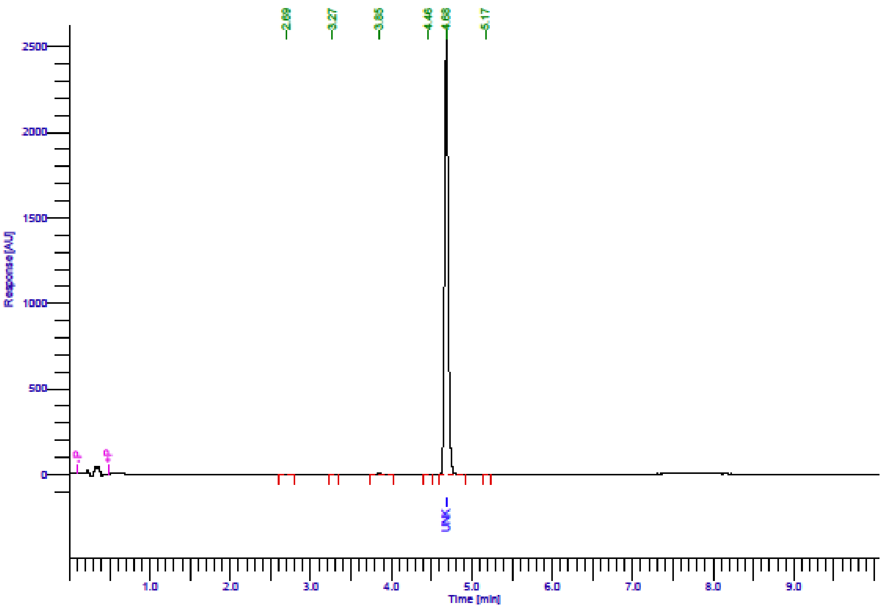


20% to 95% gradient GEMINI 2.0 ml/min Acn/H20

Wavelength 225 BW15nm, 5ul Inj

| Peak # | Time [min] | Area [uV*sec] | Height [uV] | Area [%] | Norm. Area [%] | BL | Area/Height [sec] |
|---|---|---|---|---|---|---|---|
| 1 | 2.695 | 8076.21 | 2256.64 | 0.11 | 0.11 | BB | 3.5789 |
| 2 | 3.267 | 7405.50 | 3552.03 | 0.10 | 0.10 | BB | 2.0849 |
| 3 | 3.847 | 29025.13 | 8785.48 | 0.38 | 0.38 | BB | 3.3038 |
| 4 | 4.463 | 8749.26 | 3031.38 | 0.11 | 0.11 | BB | 2.8862 |
| 5 | 4.683 | 7572568.20 | 2.54e+06 | 99.27 | 99.27 | BB | 2.9762 |
| 6 | 5.170 | 2179.70 | 1014.25 | 0.03 | 0.03 | BB | 2.1491 |
| | | 7628004.00 | 2.56e+06 | 100.00 | 100.00 | | |

Warning -- Signal level out-of-range in peak

Missing Component Report
Component Expected Retention (Calibration File)

All components were found

**Figure S10.** HPLC trace for compound **5**.

### 2-amino-4-{2,4-dichloro-5-[2-(dimethylamino)ethoxy]phenyl}-N-(2,2,2-trifluoroethyl)thieno[2,3-d]pyrimidine-6-carboxamide (1)

Compound **1** , off-white powder: LCMS $t_R$ = 0.889 min; *m/z* = 510, 508 $[M+H]^+$; $^1$H NMR (400 MHz, DMSO-d6) δ 2.23 (s, 6H), 2.68 (t, J = 5.6 Hz, 2H), 3.98 – 4.13 (m, 2H), 4.18 (t, J = 5.6 Hz, 2H), 7.36 (s, 2H), 7.42 (s, 1H), 7.76 (s, 1H), 7.84 (s, 1H), 9.17 (t, J = 6.3 Hz, 1H); $^{19}$F NMR (376 MHz, DMSO-$d_6$) δ -70.49; $^{13}$C NMR (100.6 MHz, DMSO-d6) δ 40.0 (CH2), 45.6 (CH3), 57.3 (CH2), 67.9 (CH2), 115.3 (CH), 121.1 (C), 122.3 (C), 123.1 (C), 123.1 (CH), 124.6 (C), 130.4 (CH), 130.4 (C), 135.5 (C), 152.9 (C), 161.3 (C), 161.8 (C), 162.0 (C), 171.4 (C); HRMS, calcd for $C_{19}H_{19}Cl_2F_3N_5O_2S$ $[M+H]^+$ 508.0589 found 508.0583; HPLC (*method B*) 99.8% ($t_R$ = 0.889 min).

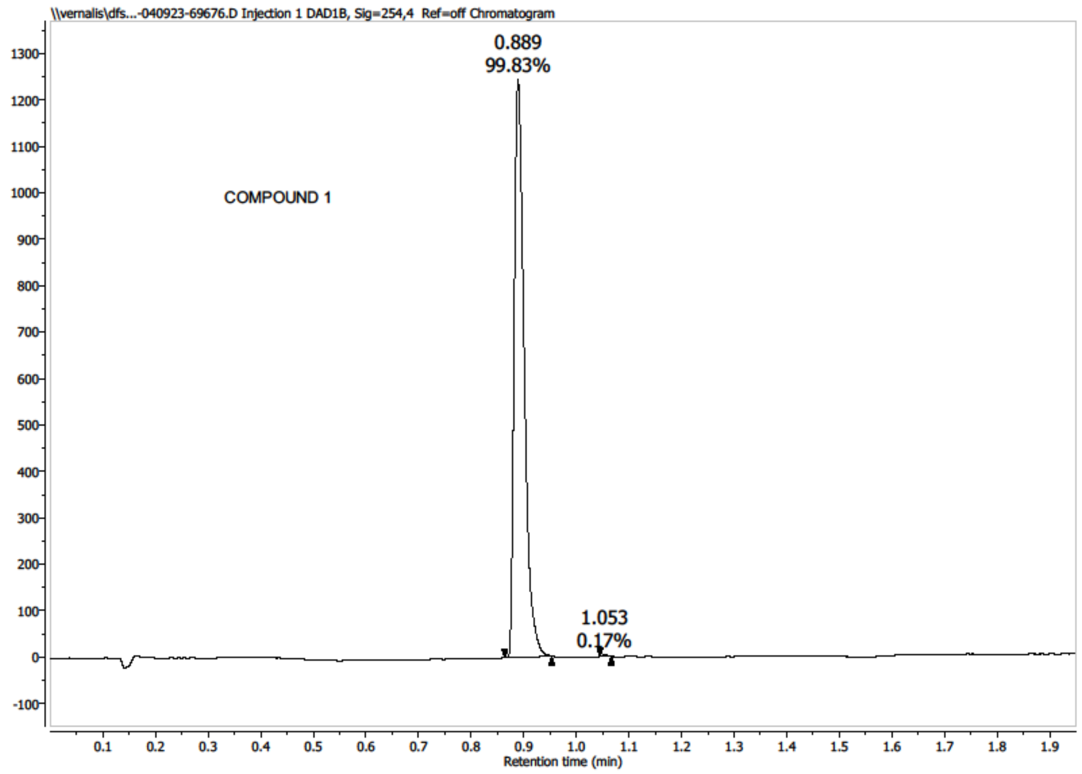


**Figure S11.** HPLC trace for compound **1**.

Compounds **2** and **6**

The synthesis and analytical methods for pertaining to compounds **6** are described in detail elsewhere.[2] Compound **2** is made and analyzed by a directly analogous method.
Compounds **2** and **6** are prepared by the synthetic procedures outlined in Scheme S2.

**Scheme S2.** Synthetic procedure utilized for preparation 4-aryl-5-cyanopyrrolo[2,3-d] pyrimidine Hsp90 inhibitors[a]

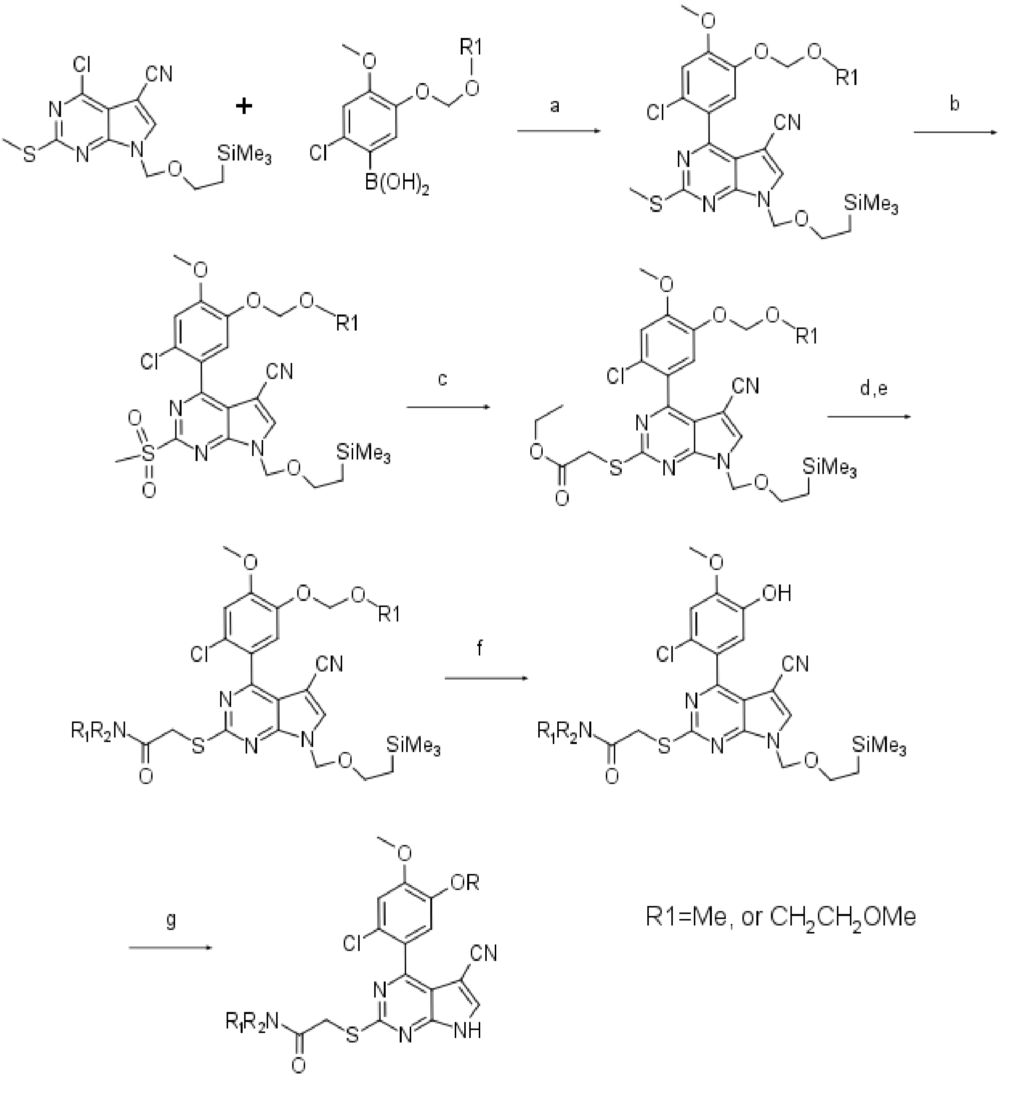


[a]Reagents and conditions: (a) $PdCl_2(PPh_3)_2$, $NaHCO_3$, DMF, 80°C, 1.5 hr, 98%; (b) *m*CPBA, $CH_2Cl_2$, 0°C to rt, 1 hr, 80%; (c) ethyl thioglycolate, NaH, THF, 0°C to rt, 1 hr, 96%; (d) NaOH (aq), rt, 2 hr; (e) $HNR_1R_2$, HBTU, MeCN, rt; (f) PPTS, *i*-PrOH, 85°C, 16 hr; (g) (i) RI, $Cs_2CO_3$, DMF, rt, 2 hr and/or (ii) TBAF, $H_2N(CH_2)_2NH_2$, THF, 40°C

Compound **6**, colorless solid: LCMS (*method A*) $t_R$ = 1.99 min; $m/z$ = 434, 432 $[M+H]^+$; $^1H$ NMR (400 MHz, $CD_3OD$) δ 2.96 (s, 3H), 3.20 (s, 3H), 3.86 (s, 3H), 3.91 (s, 3H), 4.22 (s, 2H), 7.09 (s, 1H), 7.13 (s, 1H), 8.10 (s, 1H); $^{13}C$ NMR (100.6 MHz, $CD_3OD$) δ 34.4 (CH2), 36.5 (CH3), 38.2 (CH3), 56.8 (CH3), 56.8 (CH3), 86.5 (C), 114.0 (CH), 114.4 (C), 115.1 (CH), 115.4 (C), 125.8 (C), 128.0 (C), 137.6 (CH), 149.4 (C), 152.5 (C), 154.4 (C), 159.2 (C), 166.1 (C), 170.9 (C); HRMS, calcd for $C_{19}H_{19}ClN_5O_3S$ $[M+H]^+$ found 432.0887 requires 432.0879; HPLC (*method B*) 100 % ($t_R$ = 1.04 min).

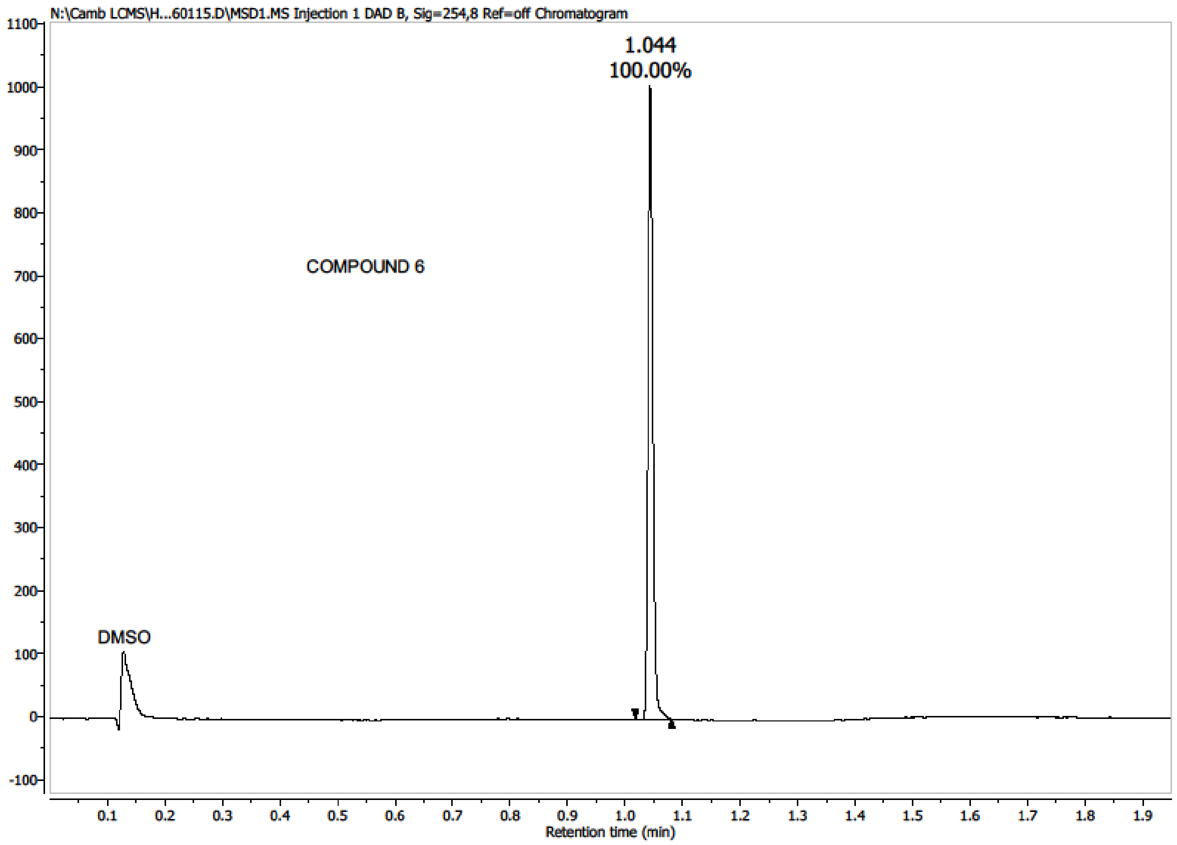


**Figure S12.** HPLC for compound **6**.

**2-{[4-(2-chloro-5-hydroxy-4-methoxyphenyl)-5-cyano-7H-pyrrolo[2,3-d]pyrimidin-2-yl]sulfanyl}-N-(2,2,2-trifluoroethyl)acetamide (2)**

Compound **2**, colorless solid: LCMS (*method A*) $t_R$ = 0.875 min; *m/z* = 472 [M+H]$^+$; $^1$H NMR (400 MHz, $CD_3OD$) δ 3.90 (q, 2H, *J* = 9.4 Hz), 3.94 (s, 3H), 4.01 (s, 2H), 6.91 (s, 1H), 7.10 (s, 1H), 8.11 (s, 1H); $^{13}$C NMR (100.6 MHz, $CD_3OD$) δ 35.5 (CH2), 41.7 (CH2), 56.8 (CH3), 86.9 (C), 113.8 (CH), 114.5 (C), 115 (C), 118.1 (CH), 124.1 (C), 125.7 (C), 128.3 (C), 137.1 (CH), 146.9 (C), 151.2 (C), 153.9 (C), 159.6 (C), 165.8 (C), 172.1 (C); HRMS, calcd for $C_{18}H_{14}ClF_3N_5O_3S$ [M+H]$^+$ found 472.0452 requires 472.0458; HPLC (*method B*) 99.4 % ($t_R$ = 0.875 min).

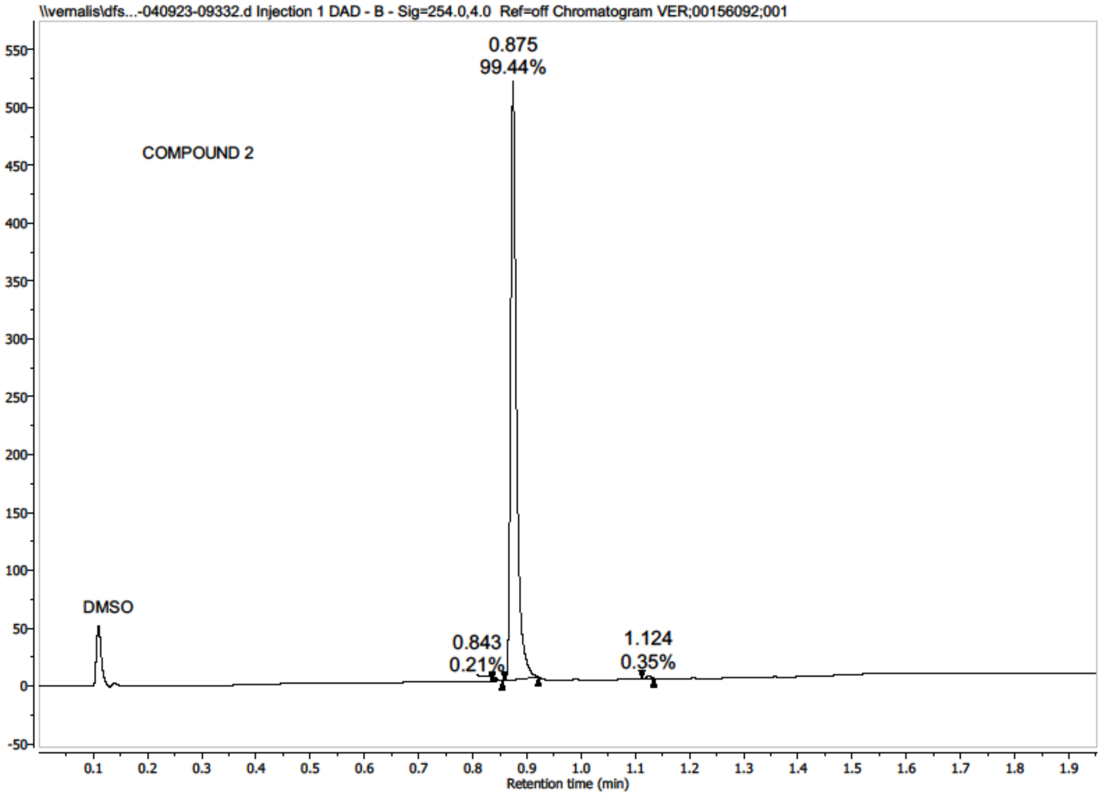


**Figure S13.** HPLC for compound **2**.

Compounds **4** and **7**

The synthesis and analytical methods for pertaining to compound **7** are described in detail elsewhere.[3]

Compound **4** is synthesized and analyzed by a directly analogous method to compound **7.**

Compounds **4** and **7** are prepared by the synthetic procedures outlined in scheme S3.

**Scheme S3.** Synthetic procedure utilized for preparation 4,5-diaryl isoxazole Hsp90 inhibitors[a]

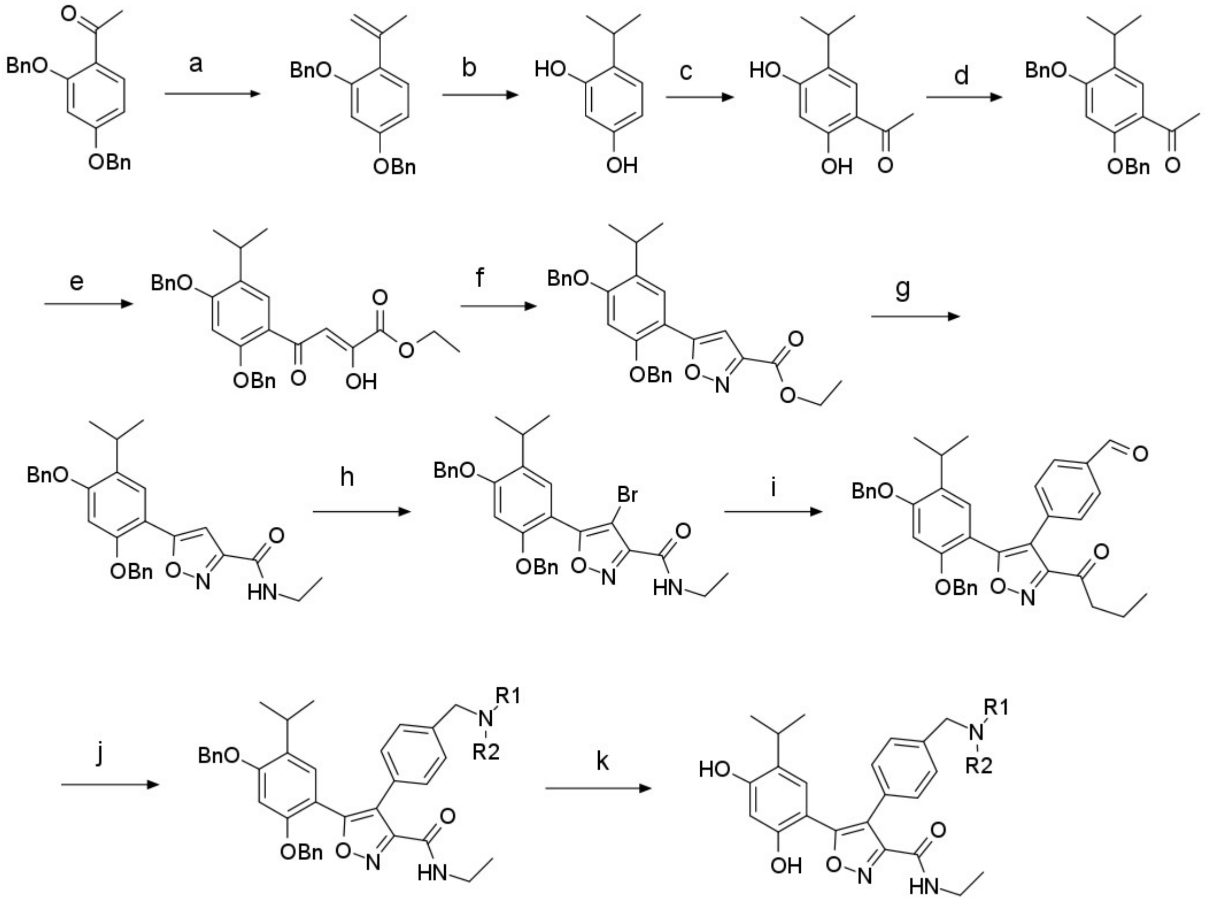


[a]Reagents and conditions: (a) $PPh_3PMeBr$, nBuLi, THF, 80%; (b) $H_2$, Pd/C, EtOH, 70%; (c) AcOH, $BF_3$•$OEt_2$, 88%; (d) BnBr, $K_2CO_3$, DMF, 88%; (e) $(CO_2Et)_2$, NaOEt, NaH, toluene, Δ, 86%; (f) Hydroxylamine hydrochloride, EtOH, Δ, 91%; (g) $EtNH_2$, MeOH, Δ, 85%; (h) NBS CAN, MeCN, Δ; (i) 4-formylphenylboronic acid, $NaHCO_3$, $H_2O$, DMF, $Pd(PPh_3)_2Cl_2$; (j) R1R2NH, $NaCNBH_3$; (k) $BCl_3$, DCM, 0°C.

**5-(2,4-Dihydroxy-5-isopropyl-phenyl)-4-(4-morpholin-4-ylmethyl-phenyl)-isoxazole-3-carboxylic acid ethylamide (7).**

Colourless solid. LCMS $t_R$ = 1.99 min., $m/z$ = 466.3 $[M+H]^+$; $^1H$ NMR (400 MHz, DMSO-$d_6$) δ 0.90 (d, 6H, $J$ = 7.1 Hz), 1.07 (t, 3H, $J$ = 7.4 Hz), 2.33 (brm, 4H), 2.97 (sept, 1H, $J$ = 7.1 Hz), 3.22 (m, 2H), 3.42 (s, 2H), 3.55 (brm, 4H), 6.43 (s, 1H), 6.72 (s,1H), 7.18 (d, 2H, $J$ = 8.4 Hz), 7.23 (d, 2H, $J$ = 8.4 Hz), 8.83 (brt, 1H, $J$ = 5.9 Hz), 9.65 (s, 1H), 9.76 (s, 1H); $^{13}C$ NMR (100.6 MHz, DMSO-$d_6$) δ 14.4 (CH3), 22.4 (CH3), 25.4 (CH), 33.7 (CH2), 53.1 (CH2), 62.1 (CH2), 66.2 (CH2),102.7 (CH), 104.5 (C), 114.7 (C), 125.6 (C), 127.7 (CH), 128.5 (C), 128.7 (CH), 128.8 (CH), 136.9 (C), 154.7 (C), 157.4 (C), 157.8 (C), 159.8 (C), 166.3 (C); HRMS, calcd for $C_{26}H_{31}N_3O_5$ $[M+Na]^+$ 488.2161, found 488.2162; HPLC (method B) 98.8% ($t_R$ = 1.28 min).

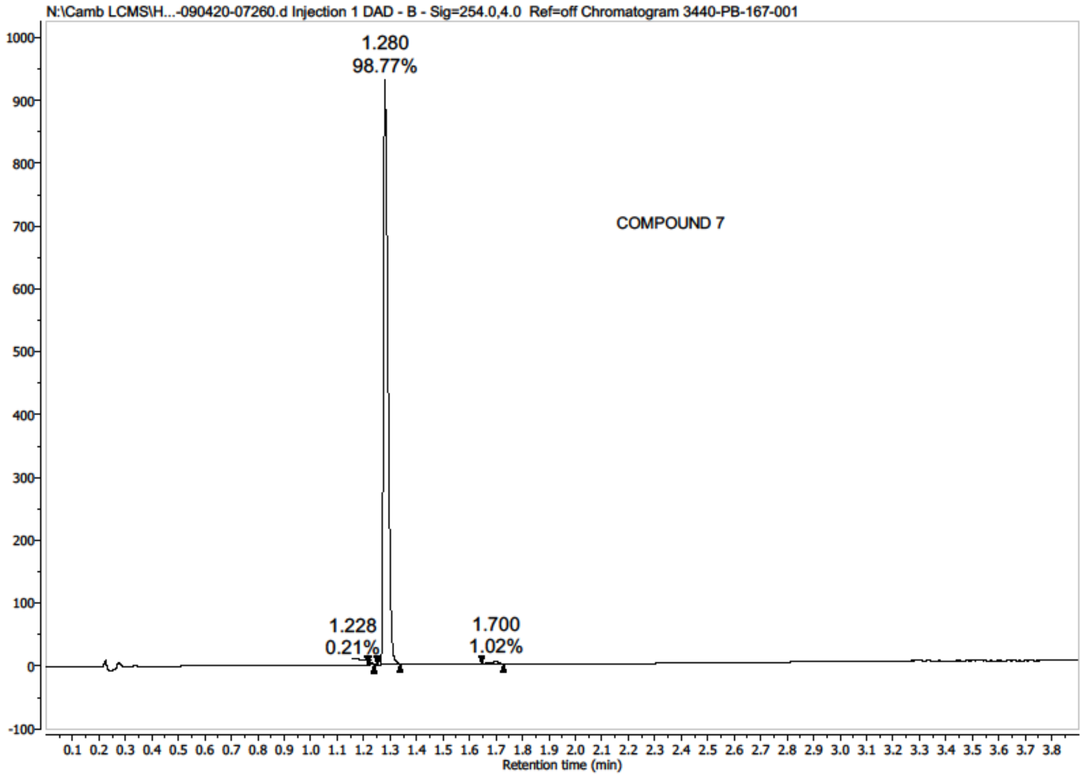


**Figure S14.** HPLC for compound **7**.

**5-[2,4-dihydroxy-5-(propan-2-yl)phenyl]-N-ethyl-4-(4-{[methyl(2,2,2-trifluoroethyl)amino]methyl}phenyl)-1,2-oxazole-3-carboxamide** (**4**).

Colourless solid. LCMS $t_R$ = 1.217 min., *m/z* = 492.2 $[M+H]^+$; $^1$H NMR (400 MHz, DMSO-d6) δ 0.92 (d, J = 6.9 Hz, 6H), 1.07 (t, J = 7.2 Hz, 3H), 2.30 (s, 3H), 2.97 (hep, J = 6.9 Hz, 1H), 3.17 – 3.29 (m, 4H), 3.68 (s, 2H), 6.44 (s, 1H), 6.74 (s, 1H), 7.17 – 7.28 (m, 4H), 8.82 (t, J = 5.7 Hz, 1H), 9.64 (s, 1H), 9.75 (s, 1H); $^{19}$F NMR (376 MHz, DMSO-$d_6$) δ -67.94; $^{13}$C NMR (100.6 MHz, DMSO-d6) δ 14.3 (CH3), 22.3 (CH3), 25.4 (CH), 33.7 (CH2), 42.2 (CH3), 56.0 (CH2), 61.0 (CH2), 102.7 (CH), 104.5 (C), 114.6 (C), 125.6 (C), 126.2 (C), 127.7 (CH), 128.2 (CH), 128.6 (C), 128.9 (CH), 137.3 (C), 154.7 (C), 157.4 (C), 157.8 (C), 159.8 (C), 166.2 (C); HRMS, calcd for $C_{25}H_{29}F_3N_3O_4$ $[M+H]^+$ 492.2110, found 492.2107; HPLC (method B) 100% ($t_R$ = 1.217 min).

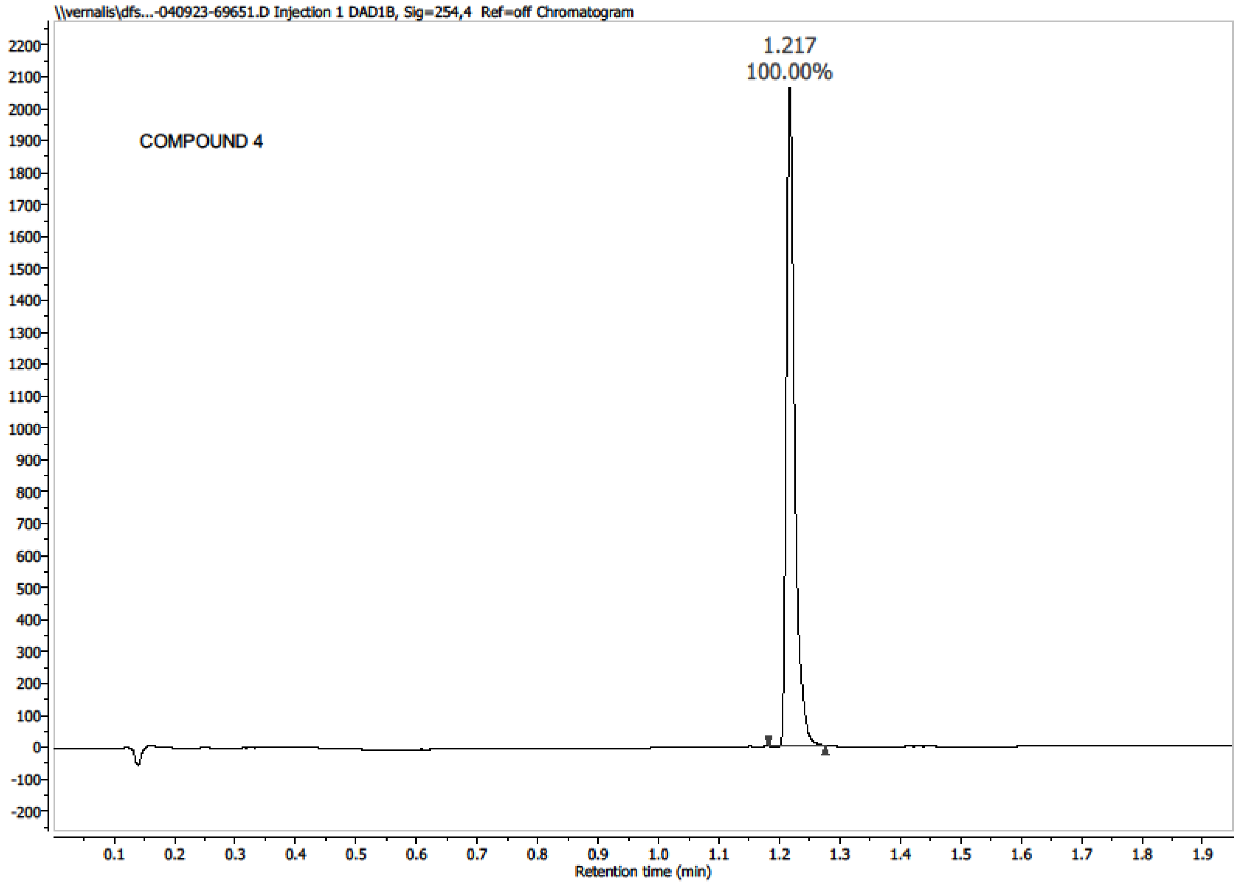


**Figure S15.** HPLC for compound **4**.

**Table S4.** Compound characterization

| Cmpd No | LCMS | $^{1}$H NMR | $^{19}$F NMR | $^{13}$C NMR | HRMS | HPLC |
|---|---|---|---|---|---|---|
| **1** | √ | √ | √ | √ | √ | √ (99.8%) |
| **2** | √ | √ | x | √ | √ | √ (99.4%) |
| **3** | √ | √ | √ | √ | √ | √ (97.3%) |
| **4** | √ | √ | √ | √ | √ | √ (100%) |
| **5** | √ | √ | x | √ | √ | √ (99.6%) |
| **6** | √ | √ | x | √ | √ | √ (100%) |
| **7** | √ | √ | x | √ | √ | √ (98.8%) |

**References**


(1) Brough, P. A.; Barril, X.; Borgognoni, J.; Chene, P.; Davies, N. G. M.; Davis, B.; Drysdale, M. J.; Dymock, B.; Eccles, S. A.; Garcia-Echeverria, C.; Fromont, C.; Hayes, A.; Hubbard, R. E.; Jordan, A. M.; Jensen, M. R.; Massey, A.; Merrett, A.; Padfield, A.; Parsons, R.; Radimerski, T.; Raynaud, F. I.; Robertson, A.; Roughley, S. D.; Schoepfer, J.; Simmonite, H.; Sharp, S. Y.; Surgenor, A.; Valenti, M.; Walls, S.; Webb, P.; Wood, M.; Workman, P.; Wright, L. Combining Hit Identification Strategies: Fragment-Based and in Silico Approaches to Orally Active 2-Aminothieno[2,3-d]Pyrimidine Inhibitors of the Hsp90 Molecular Chaperone. *J Med Chem* **2009**, *52* (15), 4794–4809. https://doi.org/10.1021/jm900357y.

(2) Davies, N. G. M.; Browne, H.; Davis, B.; Drysdale, M. J.; Foloppe, N.; Geoffrey, S.; Gibbons, B.; Hart, T.; Hubbard, R.; Jensen, M. R.; Mansell, H.; Massey, A.; Matassova, N.; Moore, J. D.; Murray, J.; Pratt, R.; Ray, S.; Robertson, A.; Roughley, S. D.; Schoepfer, J.; Scriven, K.; Simmonite, H.; Stokes, S.; Surgenor, A.; Webb, P.; Wood, M.; Wright, L.; Brough, P. Targeting Conserved Water Molecules: Design of 4-Aryl-5-Cyanopyrrolo[2,3-d]Pyrimidine Hsp90 Inhibitors Using Fragment-Based Screening and Structure-Based Optimization. *Bioorganic & Medicinal Chemistry* **2012**, *20* (22), 6770–6789. https://doi.org/10.1016/j.bmc.2012.08.050.

(3) Brough, P. A.; Aherne, W.; Barril, X.; Borgognoni, J.; Boxall, K.; Cansfield, J. E.; Cheung, K.-M. J.; Collins, I.; Davies, N. G. M.; Drysdale, M. J.; Dymock, B.; Eccles, S. A.; Finch, H.; Fink, A.; Hayes, A.; Howes, R.; Hubbard, R. E.; James, K.; Jordan, A. M.; Lockie, A.; Martins, V.; Massey, A.; Matthews, T. P.; McDonald, E.; Northfield, C. J.; Pearl, L. H.; Prodromou, C.; Ray, S.; Raynaud, F. I.; Roughley, S. D.; Sharp, S. Y.; Surgenor, A.; Walmsley, D. L.; Webb, P.; Wood, M.; Workman, P.; Wright, L. 4,5-Diarylisoxazole Hsp90 Chaperone Inhibitors: Potential Therapeutic Agents for the Treatment of Cancer. *J. Med. Chem.* **2008**, *51* (2), 196–218. https://doi.org/10.1021/jm701018h.
